# The development and implementation of a new hospital performance measure to assess hospital contributions to community health and equity

**DOI:** 10.1111/1475-6773.14018

**Published:** 2022-07-24

**Authors:** Caroline F. Plott, Rachel L. J. Thornton, Ekta Punwani, Irene Dankwa‐Mullan, Hema Karunakaram, Kelly Jean Thomas Craig, Kyu Rhee, Joshua M. Sharfstein

**Affiliations:** ^1^ Department of Medicine The Johns Hopkins University School of Medicine Baltimore Maryland USA; ^2^ Office of Health Equity and Inclusion Nemours Children's Health Wilmington DE USA; ^3^ Department of Pediatrics The Johns Hopkins University School of Medicine Baltimore Maryland USA; ^4^ Center for Health Equity Johns Hopkins Bloomberg School of Public Health Baltimore Maryland USA; ^5^ IBM Watson Health Cambridge Massachusetts USA; ^6^ Center for AI, Research, and Evaluation IBM Watson Health Cambridge Massachusetts USA; ^7^ CVS Health Woonsocket Rhode Island USA; ^8^ Department of Health Policy and Management Johns Hopkins Bloomberg School of Public Health Baltimore Maryland USA

**Keywords:** community health, health care reporting, health equity, hospital quality, measures, methods

## Abstract

**Objective:**

To develop and implement a measure of how US hospitals contribute to community health with a focus on equity.

**Data Sources:**

Primary data from public comments and hospital surveys and secondary data from the IBM Watson Top 100 Hospitals program collected in the United States in 2020 and 2021.

**Study Design:**

A thematic analysis of public comments on the proposed measure was conducted using an iterative grounded approach for theme identification. A cross‐sectional survey of 207 hospitals was conducted to assess self‐attestation to 28 community health best practice standards in the revised measure. An analysis of hospital rankings before and after inclusion of the new measure was performed.

**Data Collection/Extraction Methods:**

Public comment on the proposed measure was collected via an online survey, email, and virtual meetings in 2020. The survey of hospitals was conducted online by IBM in 2021. The analysis of hospital ranking compared the 2020 and 2021 IBM Watson Top 100 Hospitals program results.

**Principal Findings:**

More than 650 discrete comments from 83 stakeholders were received and analyzed during measure development. Key themes identified in thematic analysis included equity, fairness, and community priorities. Hospitals that responded to a cross‐sectional survey reported meeting on average 76% of applicable best practice standards. Least met standards included providing emergent buprenorphine treatment for opioid use disorder (53%), supporting an evidence‐based home visiting program (53%), and establishing a returning citizens employment program (27%). Thirty‐seven hospitals shifted position in the 100 Top Hospital rankings after the inclusion of the new measure.

**Conclusions:**

There is broad interest in measuring hospital contributions to community health with a focus on equity. Many highly ranked hospitals report meeting best practice standards, but significant gaps remain. Improving measurement to incentivize greater hospital contributions to community health and equity is an important priority.

**What is known on this topic**

- There is growing appreciation of the unique role of hospitals in addressing health disparities by improving the health of their surrounding communities.
- Investments in improving community health and in addressing social determinants complement efforts to provide quality and equitable care.
- No leading hospital evaluation or ranking programs have incorporated a set of community health measures prior to this work.

**What this study adds**

- The development and implementation of a new survey‐based instrument to measure how hospitals contribute to community health, with a focus on equity, drew broad interest, and engagement.
- Implementation of the new measure demonstrated that a majority of top hospitals are engaging in activities that promote community health, but there remain significant opportunities for further progress.
- There is a need for further development of tools to assess the impact of hospital contributions to community health and health equity.

## 1 INTRODUCTION

Over the last decade, the United States has experienced both escalating health care costs and a plateau and decline in life expectancy, with the persistence of significant racial and socioeconomic disparities in health and health care.^1^, ^2^, ^3^, ^4^ More than a century of research demonstrates that social and economic conditions in communities where people live, work, learn, and play are key drivers of population‐level health outcomes and, by extension, health disparities.^5^, ^6^ Recognizing this dilemma, hospitals are increasingly looking beyond their walls to make contributions to population health and health equity.^7^, ^8^, ^9^, ^10^

Hospital ranking and recognition programs are early in the process of adjusting to these changes. The American Hospital Association's Foster McGaw Prize uses a non‐standardized approach to recognize hospitals for community service.^7^ The Lown Index aims to rank hospitals by their social responsibility using publicly available data related to civic leadership, value of care, and community benefits.^8^ Leading hospital performance evaluation methodologies, including US News and World Report and Leapfrog, however, have remained largely focused on patient outcomes, finances, and patient experience.^9^, ^10^, ^11^

The IBM Watson Health 100 Top Hospitals study (hereto referred as 100 Top study) has assessed US hospitals for the last 28 years. This national quantitative study evaluates short‐term, non‐federal, acute care hospitals by utilizing publicly available data to assess hospital performance in five domains: inpatient outcomes, extended outcomes, operational efficiency, financial health, and patient experience.^9^, ^12^ A composite score derived from 11 outcome measures in those five domains is used to identify top‐performing hospitals that meet the inclusion criteria. The 100 hospitals in the 100 Top study are determined by ranking hospitals relative to their comparison groups: major teaching hospitals, teaching hospitals, large community hospitals, medium community hospitals, and small community hospitals.

IBM Watson Health collaborated with Johns Hopkins faculty from the Center for Health Equity and the Bloomberg American Health Initiative to create a hospital measure that recognizes the ways that health care organizations improve the health of their communities. The measure comprises hospital best practices that contribute to community health in the realms of clinical care, health‐related activities, and business practices. This novel examination of community health, with an emphasis on health equity, in a hospital performance methodology was introduced with the release of the 2021 100 Top study.

## 2 STUDY METHODS AND DATA

### 2.1 Developing the measure

The proposed hospital measure was developed in three stages: drafting, public comment, and revision.

In the first stage of drafting a new measure, the research team at the Johns Hopkins Center for Health Equity and Bloomberg American Health Initiative considered different conceptual models, including both outcome and process‐based approaches. The initial draft sought to combine these approaches, combining measures of health improvement at the county level with assessment of specific steps that hospitals can take to improve the health of their communities. The draft measure included four domains, each worth one point. The first domain, related to health improvement, awarded one point if the hospital was in the top tertile for population health outcomes in the hospital's home county as measured by the decanal trend in life expectancy or years of life lost before the age of 75. The three remaining points were based on meeting best practice standards in these domains:

1. *Hospital as health care provider (1 point)*: Whether the hospital attested to at least half of 12 best practice standards for providing clinical care that improves community health.
2. *Hospital as community partner (1 point)*: Whether the hospital attested to at least half of eight best practice standards for health‐related activities that take place in communities.
3. *Hospital as anchor institution (1 point)*: Whether the hospital attested to at least half of eight best practice standards for business practices relevant to community health.

To identify these standards, the Johns Hopkins team searched peer‐reviewed and gray literature for hospital practices that were related to leading measures of community health and:

1. aligned with existing research regarding program effectiveness to promote community health;
2. had been endorsed by leading health and medical organizations; and
3. had been implemented by hospitals in the United States.

In the second stage of measure development, the Johns Hopkins team sought public comment on the draft proposal. Invitations to comment were sent to state hospital associations; experts in the fields of medicine, hospital administration, public health, and health care and social policy; and to community‐based organizations, health care organizations, and nonprofits.

Comments on the initial proposal were submitted through (1) an online survey (Survey Monkey), which prompted participants to rank their agreement with inclusion of each best practice using a Likert scale (1 = strongly disagree to 5 = strongly agree) and also to include free‐text comments about the proposed best practices; (2) email submissions; and (3) webinar sessions and video or telephone conferences with key stakeholders conducted by members of the academic team. The public comment period lasted for 44 days, from August 13, 2020, through September 25, 2020.

Survey response scores of support for each proposed best practice were aggregated and averaged to produce individual best practice and component‐specific scores (Supplementary Exhibit 1).

In the third stage of measure development, the Johns Hopkins team used the public comments to revise the draft measure. The team grouped all of the discrete comments received into key themes and considered additional proposed best practices submitted by stakeholders. All stakeholder feedback (i.e., survey responses, emailed comments, and contemporaneous notes from webinars and meetings) was loaded into a database (Knack, version 3) and tagged by topic area. The comments were sorted by topic and grouped according to thematic content. In addition to identifying critiques or recommendations specific to proposed best practices, the analytic process identified cross‐cutting themes or recurring related ideas that applied to all or multiple domains of the proposed measure. The team arrived at a consensus around theme identification through an iterative process.

The proposed measure domains and best practices were then assessed in light of the public input to determine whether they needed to be clarified, expanded, or removed. Stakeholder recommendations for addition of new best practice standards were evaluated to determine whether they met the criteria for inclusion in the proposed measure. Each change was determined through a consensus process among the Johns Hopkins research team. A comprehensive document outlining changes to the proposed measure and other responses was posted on the Johns Hopkins Bloomberg School of Public Health American Health Initiative website along with a revised and final proposal to the IBM Watson Health 100 Top study^13^, ^14^ (Supplementary Exhibit 2).

### 2.2 Survey of hospitals

In March 2021, IBM Watson Health surveyed the 207 highest scoring hospitals from the 2019 100 Top study dataset, which was publicly available (seven hospital scores were tied). The survey provided an opportunity for hospitals to attest to the best practice standards for the three domains included in the final Hopkins proposal: (1) hospital as a provider; (2) hospital as a partner; and (3) hospital as an anchor institution. The survey also included the option for hospitals without obstetrics facilities to mark obstetrics specific questions as not applicable and to provide links to relevant websites for more information. For hospitals that were unable to be contacted by email, the administrators for the 100 Top study attempted contact by phone.

Survey data were analyzed using Stata.^15^ The analysis included the number of best practice standards in each domain attested to by each hospital and whether there were significant differences across hospitals by the following hospital characteristics: region of the country; type of community served (i.e., urban vs. rural); academic status; health system status; peer group (major teaching hospital, teaching hospital, small community, medium community, large community); ownership (governmental, proprietary, nonprofit); and payer mix. Payer mix reflected the percent of hospital days paid for by Medicare and Medicaid based on a binary variable of whether the hospital exceeded the median value for each (median Medicare payer percent was 30.7% and median Medicaid payer percent was 6.6%).

Exploratory analyses using chi‐square tests examined whether there were statistically significant differences in reported best practices among responding hospitals across each of the hospital characteristics described. Statistical significance was determined using a *p*‐value significance of less than 0.05.

### 2.3 Measure implementation

IBM Watson Health used the results from the best practices survey in the 2021 hospital rankings. For this first year, the measure was applied to the set of hospitals that had the highest 100 scores by the other 11 outcome measures. These hospitals were then reordered within their hospital peer group based on the inclusion of the new measure at equal weight to the original 11 measures.

To make this calculation, IBM Watson Health gave up to four points to each hospital for this new measure. The submission of a survey and data sharing earned 1 point for data transparency (25% of total score), and the other components of the survey (i.e., hospital as a provider, hospital as a partner, and hospital as an anchor) were each worth 1 point (25%) if the hospital attested to at least half of the best practice standards listed in each component respectively.

Final results from the ranking were published publicly on April 27, 2021 by Fortune Magazine and IBM Watson Health.^16^, ^17^, ^18^

## 3 RESULTS

### 3.1 Developing the measure: Public engagement process

The proposed draft measure of community health with a focus on equity received over 650 discrete comments from over 83 commentators, the majority of whom were hospital or health systems, nonprofit organizations, and universities (Table 1). Comments were received via 68 online survey responses, over 19 emails, and 15 virtual meetings.

**TABLE 1:** Organizational affinities of responder hospitals

Category	Percent (*n*)
Hospital/Health System	41% (28)
Nonprofit	19% (13)
Education/University	6% (4)
Hospital Association	6% (4)
School of Public Health	4% (3)
Dept of Health	3% (2)
Community organization	2% (1)
Health data services	2% (1)
Health plan	2% (1)
Individual	2% (1)
Specialty society	2% (1)
N/A	12% (8)
Total	100% (67)

*Note*: Organizational affinities of responders to the online survey during the public comment period of the measure development. These affinities are self‐identified, and not all commenters provided an affinity (N/A).

Many of the comments were supportive of the proposed measure. On average, support for the proposed best practice standards was high (survey score of 4.4 of 5). Qualitative comments argued that hospitals have an important role to contribute to community health and that measurement of these contributions should be included in hospital ranking systems. For example, one comment praised that the measure highlights “the important role of hospitals as key community stakeholders.”

Five overarching themes emerged in the comments: equity, fairness, community priorities, measurement and scoring, and relationship with community benefit.

#### 3.1.1 Equity

Some respondents called for more attention to equity in health care delivery, arguing that community health measures should more explicitly assess whether hospitals' actions impact equitable health care delivery or access to care for minoritized populations, including non‐white racial and ethnic groups, low‐socioeconomic status populations, patients with preferred health care language other than English, LGBTQ populations, and rural populations. In response, the Johns Hopkins team response acknowledged the importance of these points, and then distinguished the goal of the new measure—to assess hospital contributions to improving the overall health of the surrounding community, including community populations not receiving health care services within the hospital walls—from the goal of evaluating whether health care services and patient outcomes are equitable. Equity in health care delivery is a distinct and critical area where hospital ranking systems should focus future measurement efforts.

Some respondents commented that more attention should be paid to racial justice considerations across the proposed metrics. One comment stated, “As applicable, all of these standards should seek to assess relevant equity gaps and measure closure of these gaps by race/ethnicity and income.”

Other comments pointed out challenges related to measuring disparities between groups directly, including the availability and reliability of data and the difficulty in identifying measures that work across different types of hospitals and communities. For example, some comments noted that the assessment of disparities may be particularly challenging in communities where the population is less racially diverse.

In response to these comments, the Johns Hopkins team supported the further development and adoption of metrics and standards that could increase accountability for reducing disparities in health and health care, including through the use of explicit measures of racial equity. Fundamentally, an equity‐driven approach requires attention to populations of greatest need, including historically marginalized populations.

#### 3.1.2 Fairness

Some respondents raised concerns about the fairness of the proposed measure. For example, one stated, “I think it's unfair for health systems and hospitals alone to solve societal problems.” Another comment stated that the “amount a health system can contribute to the success of these metrics may vary depending on a number of factors, including resources.” Other fairness concerns included questioning whether it was appropriate to apply one set of standards to the entire hospital industry.

At the same time, many respondents noted ways in which hospitals can contribute to community health, with one comment recognizing that it is important for hospitals to “be accountable for their communities…beyond health fairs and covering charity care.” Another stated, “We appreciate the basic concept of the framework – that is, to reflect the enormous efforts that hospitals and health systems undertake to contribute to the health and well‐being of their communities, and to reduce health care disparities.”

#### 3.1.3 Community priorities

Some respondents called for more attention to self‐identified community needs, with one saying, “We do not take issue with the standards proposed; however, there is risk in prescribing standards without knowing the unique needs of the community in which the standards would be applied.”

In response to this comment, the Johns Hopkins team noted it would not be feasible for a ranking system to assess community activities of individual hospitals, ascertain the priorities of each community, and compare the two. It was noted that the community health issues covered by the best practice standards are broadly relevant to diverse US communities, and meeting all the standards proposed would not be required for full credit. In addition, the Johns Hopkins team modified the proposed best practice standard on community health needs assessment to require the input of communities in this process.

#### 3.1.4 Measurement and scoring

Several respondents questioned the utility of hospitals' self‐reporting implementation of best practice standards while acknowledging the lack of other publicly available data for independent assessment. In response, the Johns Hopkins team encouraged public disclosure of hospital attestation to the best practice standards with the option for hospitals to include a link to more information about each of their programs. This practice can encourage local organizations to engage hospitals in self‐reflective conversations within their communities about efforts to improve community health.

#### 3.1.5 Relationship with community benefit

Some respondents called for greater alignment with federal community benefit requirements. One commenter stated that, “We worry about the potential for misalignment between this measurement framework, and the other ways in which hospitals are held accountable for providing community benefit…one could envision scenarios in which hospitals do not score well on this particular report card, and yet still exceed any local, state, and federal requirements around community benefit.”

The response to these comments noted that this measure is different in purpose and implementation from community benefit standards, which are broadly defined, based on tax requirements, and applicable only to nonprofit hospitals. The goal of this measure is to assess contributions to community health that are based on evidence and that reflect the diverse roles that all hospitals play in their communities.

Based on the comments provided, the Johns Hopkins team made more than 50 changes to the proposed measure. These included adding new best practice standards related to screening for alcohol use, supporting resilience among older adults, providing social needs screening and follow‐up, offering a pathway to employment for returning citizens, and encouraging environmentally sustainable practices. The final measure included 28 best practice standards across the three domains: hospital as a health care provider (12 standards); hospital as a community partner (8 standards); and hospital as an institution (8 standards) (Table 2). The specific elements needed to meet these standards are in Supplementary Exhibit 2.

**TABLE 2:** Final version of proposed hospital measure

Component 1: Community Health Metric—Decanal trend in life expectancy or years of potential life lost and trend in preventable hospitalizations (Must be in top tertile to receive credit)
Component 2: Hospital as Provider (Must meet ≥ six standards for credit)
2.1 Our hospital is a comprehensive tobacco‐free campus.
2.2 Our hospital has an inpatient tobacco use cessation program.
2.3 Our hospital provides buprenorphine treatment for opioid use disorder in the emergency department (ED).
2.4 Our Hospital provides screening, brief intervention, and referral to treatment for alcohol use in the ED and hospital.^a^
2.5 Our hospital runs a hospital‐based violence prevention program.
2.6 Our hospital screens for intimate partner violence and refers to services and supports as needed.
2.7 Our hospital offers healthy food options.
2.8 Our hospital has a social needs screening and referral program.^a^
2.9 Our hospital offers an infant safe sleep education program.
2.10 Our hospital adopts 10 practices to support breastfeeding.^a^
2.11 Our hospital offers contraception treatment and counseling to patients immediately postpartum.^a^ ^,^ ^b^
2.12 Our hospital implements practices to reduce falls and optimize mobility for elderly patients per the Age Friendly Hospital Program.^b^
Component 3: Hospital as Community Partner (Must meet ≥ four standards for credit)
3.1 Our hospital performs a community needs assessment with the department of health.
3.2 Our hospital provides meaningful support for a community‐based hypertension control program.
3.3 Our hospital provides meaningful support for a community‐based diabetes prevention program.
3.4 Our hospital provides meaningful support for an evidence‐based home visiting program.
3.5 Our hospital provides meaningful support for training and work of community health workers.
3.6 Our hospital makes meaningful contributions to supporting school success.
3.7 Our hospital meaningfully supports expanding access to fresh, healthy foods in the community.
3.8 Our hospital invests in expanding or improving healthy, affordable housing in the community.
Component 4: Hospital as Anchor Institution (Must meet ≥ four standards for credit)
4.1 Our hospital has a 5‐year plan for achieving diversity in board and top management.
4.2 Our hospital pays all employees a minimum hourly rate based on the local living wage.
4.3 Our hospital has a minority‐owned business purchasing and procurement goal and measures progress toward this goal.
4.4 Our hospital supports access to affordable high‐quality child care for children of all full and part‐time employees.
4.5 Our hospital provides paid sick leave to all employees.
4.6 Our hospital adopts a “do no harm” collections policy.
4.7 Our hospital has a returning citizen work program.^a^
4.8 Our hospital publishes plans for advancing sustainability.^a^

*Note*: Final version of the proposed hospital measure, organized by component, with guidelines for scoring. Details for each of the best practice standards are available in Supplementary Files.

^a^ Measure that may not apply to all hospitals.

^b^ Measure that was added after the public comment period.

### 3.2 Survey of hospitals

Of the 207 hospitals surveyed by IBM Watson Health, 116 responded, 69 of which were in the top 100. The majority of respondents served urban communities as part of a health system, were located in the South or North‐Central regions of the country, and were under nonprofit ownership. Responding hospitals were more likely to see a greater percentage of Medicaid patients than the median hospital.

The hospitals reported meeting an average of 76% of applicable best practice standards across the three measure domains (i.e. hospital as [1] health care provider, [2] community partner, and [3] anchor institution). Ninety (78%) met at least half of applicable best practice standards in all three areas, 16 (14%) in two areas, seven (6%) in one area, and three (3%) in no areas (Table 3).

**TABLE 3:** Characteristics of top 200 hospitals

Criteria	% Hospitals that meet the criteria and returned a survey (*n*)	% Hospitals that meet the criteria and did not return a survey (*n*)	*p*‐value
Urban (178)	87% (101)	85% (77)	0.61
Academic (46)	40% (46)	0% (0)	n/a
Health system (101)	87% (101)	0% (0)	n/a
Region			
West (44)	23% (27)	19% (17)	0.42
Northeast (15)	5% (6)	10% (9)	0.19
South (74)	36% (42)	35% (32)	0.88
North central (74)	35% (41)	36% (33)	0.89
Control			
Nonprofit (160)	76% (88)	79% (72)	0.58
Proprietary (36)	18% (21)	16% (15)	0.76
Governmental (11)	6% (7)	4% (4)	0.60
Medicaid days above median (90)	50% (58)	35% (32)	0.03
Medicare days above median (96)	50% (58)	42% (38)	0.24
Peer group			
Small community (42)	21% (24)	20% (18)	0.87
Medium community (40)	20% (23)	19% (17)	0.84
Large community (42)	20% (23)	21% (19)	0.85
Teaching (52)	28% (32)	22% (20)	0.36
Major teaching (31)	12% (14)	19% (17)	0.19
Totals	116	91	

*Note*: Characteristics of the top 200 hospitals invited to respond to the hospital measure survey, by responder status. Data reported as the percent (*n*) of hospitals meeting the criteria in column one out of the 116 hospitals that returned a survey (column 2) or out of the 91 hospitals that did not return the survey (column 3). *p* < 0.05 was used to determine statistical significance.

#### 3.2.1 Hospitals as health care providers

Hospitals met an average of 9.6 (range: 2–12) of the applicable best practice standards in this area (12 maximum). The least commonly met standards were providing buprenorphine treatment in the emergency department (53%, 61 hospitals) and having a hospital‐based violence prevention program (66%, 76 hospitals).

A greater proportion of small community hospitals reported having a hospital‐based violence prevention program, while teaching hospitals and hospitals in the South reported having such a program less frequently. Major teaching hospitals also reported providing buprenorphine in the ED more often.

The standards that were most frequently reported as met in this category included comprehensive tobacco‐free campus (97%, 113 hospitals), encourages healthy food choices (93%, 108 hospitals), tobacco use cessation program (92%, 107 hospitals), and support for breastfeeding (91%, 93 hospitals) (Table 4).

**TABLE 4:** Association of hospital characteristics with best practice standard attestation

Component 2: Hospital as a health care provider			
Best practice standard	% hospitals credit (*n*)^a^	More likely (%, *p*‐value)	Less likely (%, *p*‐value)
Credit for Component 2	94% (109)	North‐Central location (100% vs. 91%, 0.044)	South location (86% vs. 99%, 0.005)
Comprehensive tobacco‐free campus	97% (113)	—	Northeast location (83% vs. 98%, 0.026), large community (91% vs. 99%, 0.039)
Encourages healthy food choices	93% (108)	Nonprofit‐owned (97% vs. 82%, 0.009)	Southern location (83% vs. 99%, 0.002); Government owned (71% vs. 95%, 0.02)
Tobacco use cessation program	92% (107)	—	Academic owned (85% vs. 97%, 0.015)
Supports breastfeeding	91% (93)	—	—
Infant safe sleep education	86% (110)	Nonprofit‐owned (90% vs. 75%, 0.048)	Medicare Days above median (79% vs. 93%, 0.031); Proprietary owned (71% vs. 89%, 0.030)
SBIRT for Alcohol in ED and Hospital	85% (99)	Part of health System (89% vs. 60%, 0.003)	Government owned (57% vs. 87%, 0.030)
Age Friendly Hospital Program	84% (97)	—	—
Screens and Refer for intimate partner violence	82% (95)	—	West location (63% vs. 88%, 0.004)
Social needs screening and referral program	79% (92)	Nonprofit‐owned (84% vs. 64%, 0.024)	—
Contraception treatment and counseling postpartum	77% (72)	West location (92% vs. 72%, 0.041), large teaching (100% vs. 74%, 0.036)	Medicare Days above median (67% vs. 86%, 0.013)
Hospital‐based violence prevention program	66% (76)	Small community (83% vs. 61%, 0.039)	South location (50% vs. 74%, 0.008), teaching (50% vs. 71%, 0.03)
Buprenorphine treatment in ED	53% (61)	Large teaching (79% vs. 49%, 0.038)	—

Component 3: Hospital as a community partner			
Description	% hospitals credit (*n*)^a^	More likely (%, *p*‐value)	Less likely (%, *p*‐value)
Credit for Component 3	89% (103)	—	—
Community needs assessment	93% (108)	Nonprofit‐owned (99% vs. 75%, <0.001); North‐Central location (100% vs. 89%, 0.03)	Proprietary owned (67% vs. 99%, <0.001); South location (86% vs. 97%, 0.018); teaching (84% vs. 96%, 0.022)
Support school success	91% (105)	Nonprofit‐owned (94% vs. 79%, 0.013)	—
Supports expanding access to healthy foods	85% (99)	Nonprofit‐owned (93% vs. 61%, <0.001)	South location (76% vs. 91%, 0.036); Proprietary owned (62% vs. 91%, 0.001); Government owned (57% vs. 87%, 0.03)
Support for a diabetes prevention program	77% (89)	Nonprofit‐owned (82% vs. 61%, 0.021)	Proprietary owned (57% vs. 81%, 0.019), teaching (63% vs. 82%, 0.025)
Support for training and work of community health workers	72% (84)	High Medicaid days (82% vs. 62%, 0.008)	High Medicare days (63% vs. 81%, 0.034); South location (60% vs. 80%, 0.019)
Support for hypertension control program	68% (79)	Large Teaching (93% vs. 65%, 0.034)	—
Support for healthy, affordable housing	60% (69)	Nonprofit‐owned (65% vs. 43%, 0.04)	—
Support for an evidence‐based home visiting program	53% (61)	Part of health system (56% vs. 39%, 0.031)	—

Component 4: Hospital as an institution			
Description	% hospitals credit (*n*)^a^	More likely (%, *p*‐value)	Less likely (%, *p*‐value)
Credit for Component 4	84% (97)	—	South location (74% vs. 89%, 0.031)
Paid sick leave to all employees	91% (105)	—	—
“Do no harm” collections policy	88% (102)	—	—
Plan for advancing sustainability	71% (82)	—	Northeast‐located (33% vs. 73%, 0.039)
Minimum hourly wage	70% (81)	—	West‐located (52% vs. 75%, 0.02), Nonprofit‐owned (65% vs. 86%, 0.035)
Diversity Plan and Progress	68% (79)	Part of health system (73% vs. 33%, 0.002); West‐located (85% vs. 63%, 0.03)	—
Affordable high‐quality child care	66% (76)	Large teaching (93% vs. 62%, 0.022)	Small community (46% vs. 71%, 0.023)
Minority‐owned business purchasing and procurement goal	65% (75)	Part of health system (68% vs. 40%, 0.032), medium community (83% vs. 60%, 0.044)	High Medicare days (53% vs. 76%, 0.012)
Returning citizens employment program	27% (31)	—	West‐located (11% vs. 31%, 0.036)

*Note*: The association of hospital characteristics with attestation to best practice standards for community health. Results reported are those that are statistically significant with a chi‐square test at *p* < 0.05.

Abbreviations: ED, emergency department; SBIRT, screening, brief intervention, and referral to treatment.

^a^ A total of 116 hospitals were eligible for each best practice standard, with two exceptions. One hundred two hospitals were eligible for the “supports breastfeeding” standard, and 93 were eligible for “contraception treatment and counseling post‐partum.”

#### 3.2.2 Hospitals as community partners

Hospitals reported meeting an average of 6.0 (range 0–8) of the eight best practice standards in this area. The least frequently reported standards were support for an evidence‐based home visiting program (53%, 61 hospitals); support for healthy, affordable housing (60%, 69 hospitals); and support for hypertension control (68%, 79 hospitals).

Large teaching hospitals more often reported support for a hypertension control program. Nonprofit hospitals more often supported healthy, affordable housing.

Hospitals that are part of a health system more often reported supporting an evidence‐based home visiting program. The standards that were most frequently reported as met were having a community needs assessment (93%, 108 hospitals) and supporting school success (91%, 105 hospitals) (Table 4).

#### 3.2.3 Hospitals as anchor institutions

Hospitals reported meeting an average of 5.4 (range 0–8) of the eight best practice standards in this area. The least frequently reported standards were having a returning citizens employment program (27%, 31 hospitals), having a minority‐owned business purchasing and procurement goal (65%, 75 hospitals), providing access to affordable, high‐quality childcare (66%, 76 hospitals), and having a diversity plan for improving representation in board and top management (68%, 79 hospitals).

Hospitals that are part of health systems and those in the West more often reported having a diversity plan. Hospitals in the West less often reported having a returning citizen employment program. Large teaching hospitals reported providing affordable, high‐quality childcare more often, while small community hospitals were less likely to report doing this. Medium community hospitals and hospitals that were part of health systems reported having a minority‐owned business purchasing and procurement goals more often, and hospitals with a high percent of Medicare days reported such a plan less often. Northeast‐located hospitals were less likely to report having a plan for advancing sustainability. West‐located and nonprofit‐owned hospitals were less likely to report having a living hourly wage.

The standard most frequently reported as met was offering paid sick leave to all employees (91%, 105 hospitals) (Table 4).

### 3.3 Measure implementation

Of the 69 hospitals in the top 100 that responded to the survey, 53 (77%) earned three points for meeting at least half of the applicable standards in each section, 10 (14%) received two points, 4 (6%) received one point, and 2 (3%) received zero points. Results from the survey changed the final ranking position of 37 hospitals, whereby 17 hospitals moved up in rank by an average of 1.1 positions and 20 hospitals moved down in rank by an average of 1.1 positions.

## 4 DISCUSSION

The development and implementation of a measure of hospital contributions to community health, with a focus on equity, generated substantial interest and participation. Worsening health statistics, continued health inequities, and rising costs underscore the importance of this project. The substantial participation of health systems in the discussion reflects greater appreciation by leadership of the potential for hospitals to make a difference through efforts and partnerships that extend beyond the hospital walls.

How best to measure these contributions, however, is unsettled. Publicly available data are neither specific to local health challenges nor to the potential steps that hospitals can take. At the same time, the range of potential hospital activities that might be linked to community health is quite broad. The IBM Watson measure was developed after a search of professional standards and academic literature, a public comment process, and substantial revisions. Nonetheless, there is room for disagreement over whether particular best practice standards are sufficiently clear, achievable, and related to community health and equity to merit inclusion.

The IBM Watson measure categorized hospital contributions in three domains: hospital as a clinical provider; a community partner; and an anchor institution. Among the surveyed 207 high‐performing hospitals, on average, hospitals that responded to the survey met most of the best practice standards (76%); however, sizeable gaps in implementation of some of the best practice standards highlight the uneven nature of the current state of hospital contributions to community health. For example, it is notable that despite the rising toll of drug overdoses, taking more than 100,000 lives in the last year, one of the least met standards is the provision of buprenorphine treatment in the emergency department (53%, 61 hospitals)—a practice associated with substantially increased engagement with addiction treatment services within 1 month.^19^, ^20^

Relatively few hospitals attested to supporting an evidence‐based home visiting program (53%, 61 hospitals), despite extensive and consistent evidence of the positive impact of such programs on maternal and child health outcomes, including improved cognitive and behavioral outcomes, lower rates of maternal mortality, and reduced low birth weight.^21^, ^22^ Further, just over a quarter of hospitals reported having a returning citizens employment program, despite such programs providing more employment opportunities for previously incarcerated individuals, who are disproportionately people of color, and who face more barriers when seeking employment.^23^, ^24^

One way to encourage the adoption of these and other similar practices is to appeal to the social mission of hospitals, most of which are critical community institutions with deep roots in the regions they serve. Another approach is to align financial incentives with community health outcomes, a key goal of population‐based payment reform. For example, statewide payment models in both Vermont and Maryland incentivize the achievement of community health goals, such as reductions in diabetes prevalence and fatal overdoses. Complementing these steps is recognition of major contributions to community health with a focus on equity—not as an extra credit activity, but as a core component of what it means to be a high‐performing hospital.

The 100 Top study incorporated the community contributions to health measure soon after the development. Following implementation, 37 hospitals of the top 100 had ranking adjustments as a result. Further accountability may accompany the public release of the self‐attestations for participating hospitals.

There are limitations to this new community health measure, the most significant of which stems from the use of a survey as a data collection instrument. This format is inherently biased by self‐reporting, lack of a formal verification system, and selection bias. Hospital self‐report surveys are used in several existing hospital assessment tools, including Leapfrog Rating and US News Best Children's Hospital rankings.^25^, ^26^ Future iterations of the measure may include more robust methods for increasing response rate and holding hospitals accountable for the best practices that they attest to.

The study of current hospital community health practices is also limited by the fact that only the top 200 hospitals were surveyed during the measure implementation's pilot year. It is therefore not clear the extent to which these results are representative of hospitals across the country.

Despite these limitations, this initial analysis demonstrated that measurement of hospital contributions to community health and equity is possible. It also revealed four major areas for future work. First, progress on measurement is urgently needed. One of the challenges highlighted in the development of this measure is the lack of readily available population health outcomes by race, ethnicity, or socioeconomic status at the community level. Furthermore, most national data sources are not available at the sub‐county level for health outcomes, which limits the ability of hospitals to look at impacts on their immediate geography.

Although outside the scope of this measure, the comment period also revealed tremendous interest in more comprehensive assessment of equity in the delivery of health services. There is no national source of clinical outcomes by race or ethnicity, such as readmission rates, adverse outcomes, or patient experience. Attention to these gaps should be a priority.

Second, there should be more research on the impact of specific hospital practices on community health. Many of the best practice standards included in the new measure relate both directly and indirectly to community health outcomes, but studies should assess whether hospital contributions demonstrably impact community health at scale. A curation of impact evaluations within the peer‐reviewed literature would raise certain best practices above the others. To date, the majority of research, societal guidelines, and consensus documents have focused on clinical interventions by hospitals. Evidence assessing how hospitals can positively impact community health and advance health equity in their roles as community partners or anchor institutions should be further developed.

Third, more attention is due at the interface of hospital practice and public policy. Hospitals have financial, environmental, and political impacts within their communities. Assessing such impacts should be considered for future measures of this type.

Fourth, measurements of hospital efforts should expand—beyond these initial ideas, beyond 200 high‐performing hospitals, and beyond one ranking system. Given the urgency of improving health outcomes in the United States, no comprehensive hospital performance evaluation should be complete without addressing contributions to community health with a focus on equity.

## 5 CONCLUSIONS

Broad stakeholder participation led to the creation of a hospital measure to assess contributions to community health with a focus on equity. Most leading hospitals self‐attest to many, but not all, of the 28 best practice standards incorporated in the measure. Inclusion of the measure in the 2021 100 Top study led to a substantial shift in hospital rankings. Future work should expand and improve upon this initial effort.

## FUNDING INFORMATION

This research study was supported in part by IBM Watson Health and some co‐authors are or were employed by IBM Corporation (Ekta Punwani, Irene Dankwa‐Mullan, Hema Karunakaram, Kyu Rhee). This research was also supported by the Johns Hopkins School of Medicine Dean's Research grant (Caroline F. Plott) and by the Bloomberg American Health Initiative (Joshua M. Sharfstein, Rachel L. J. Thornton). Unrelated to this work, Dr. Sharfstein reports receiving consulting fees from Sachs Policy Group, where he advises health care systems on payment reform.

## Supporting information


**Appendix S1.** Supporting information.

**Appendix S2.** Supporting information.
