# Supplementary material for: The development and implementation of a new hospital performance measure to assess hospital contributions to community health and equity
Source: Health Serv Res. 2022 Jul 24;57(Suppl 2):304–14. doi: 10.1111/1475-6773.14018 (PMC9660406; doi:10.1111/1475-6773.14018)
Supplement: Supplementary file 1 — Appendix S1. Supporting information. [file HESR-57-304-s002.docx]

**Supplementary Materials**

Supplementary Exhibit 1. Support for Proposed Draft Best Practice Standards: Mean score provided by public comment survey respondents on Likert scale, 1- strongly disagree to 5 – strongly agree. Score is out of 5.

| ***Component 2 Best Practices Average Score of Support*** | |
| --- | --- |
| Comprehensive tobacco free campus | 4.7 |
| Tobacco use cessation program | 4.3 |
| Encourages healthy food choices | 4.4 |
| Buprenorphine treatment in ED | 4.2 |
| Hospital-based violence prevention program | 4.2 |
| Screens & Refer for intimate partner violence | 4.5 |
| Infant safe sleep education | 4.6 |
| Support breastfeeding | 4.5 |
|  | |
| ***Component 3 Best Practices Average Score of Support*** | |
| Community needs assessment | 4.6 |
| Support for hypertension control program | 4.3 |
| Support for a diabetes prevention program | 4.3 |
| Support for an evidence-based home visiting program | 4.4 |
| Support for training & work of community health workers | 4.4 |
| Support school success | 4.4 |
| Supports expanding access to healthy foods | 4.5 |
| Support for healthy, affordable housing | 4.2 |
|  | |
| ***Component 4 Best Practices Average Score of Support*** | |
| Diversity Plan & Progress | 4.4 |
| Minority owned business purchasing and procurement goal | 4.4 |
| Minimum hourly wage | 4.6 |
| Affordable high-quality child care | 4.4 |
| Paid sick leave to all employees | 4.7 |
| “Do no harm” collections policy | 4.5 |
