# Supplementary material for: The development and implementation of a new hospital performance measure to assess hospital contributions to community health and equity
Source: Health Serv Res. 2022 Jul 24;57(Suppl 2):304–14. doi: 10.1111/1475-6773.14018 (PMC9660406; doi:10.1111/1475-6773.14018)
Supplement: Supplementary file 2 — Appendix S2. Supporting information. [file HESR-57-304-s001.docx]

Supplementary Exhibit 2. Measuring Hospital Contributions to Community Health with a Focus on Equity -- A Proposed Approach for the IBM Watson Health Rankings.

| **2.1. Best Practice Standard: Hospital is a comprehensive tobacco-free campus.** |
| --- |

*Best Practice Standard*. Hospitals can establish and enforce a completely tobacco-free campus as recommended by the American Medical Association (AMA). The AMA supports that “all American hospitals ban tobacco and supports working toward legislation and policies to promote a ban on smoking and use of tobacco products in, or on the campuses of, hospitals, health care institutions, retail health clinics, and educational institutions, including medical schools.”^1^ A tobacco-free campus does not allow smoking (including e-cigarettes) or use of smokeless tobacco in indoor and outdoor areas.^2,3^

*Example*. Many hospital campuses have established smoke free policies including the Veteran Health Administration (USA) and University of Wisconsin Hospital and Clinics (WI).^4,5^

*Background*. Smoking negatively contributes to almost all health conditions. Decreased rates of smoking are associated with fewer cardiovascular events and decreased asthma morbidity. Comprehensive tobacco-free policies are associated with halving secondhand smoke exposure as well as decreasing the prevalence of tobacco smoking and tobacco consumption.^6^

| **2.2. Best Practice Standard: Hospital has a tobacco use screening and cessation program that is initiated while the patient is hospitalized.** |
| --- |

*Best Practice Standard.* Hospitals can implement the strategies noted by the 2020 report of the U.S. Surgeon General on smoking cessation. These are summarized in the report’s Table 7.2 and include the following:^7^

| 1. Implement a system to identify and document the tobacco use status of all hospital patients 2. Identify a clinician(s) to deliver nicotine dependence services to inpatients at every hospital and reimburse hospitals for delivering such services 3. Offer nicotine dependence treatment to all hospital patients who use tobacco 4. Expand hospital formularies to include FDA-approved nicotine dependence medications 5. Ensure compliance with The Joint Commission’s regulations mandating that all sections of the hospital be entirely smokefree and that patients receive cessation treatments 6. Educate hospital staff about medications that may be used to reduce nicotine withdrawal symptoms, even if the patient is not intending to quit at that time   *Report of the Surgeon General, 2020* ^7^ |
| --- |

*Example*. The University of Wisconsin-Madison (WI) developed an inpatient tobacco cessation intervention which provides each patient who has reported that they smoke an option to have brief counselling and to meet with a pharmacist who can offer the patient tobacco cessation medications.^8^ This program is available regardless of the patient’s insurance status.

*Background.* The Centers for Disease Control and Prevention estimate that 480,000 people die every year as a result of tobacco product use and exposure in the US alone.^9^ Furthermore, tobacco use puts patients at increased risk of lung cancer, chronic conditions like cardiovascular disease and hypertension, and harm to the fetus during pregnancy.^10-13^ Hospitalization is an opportunity to support tobacco cessation by providing patients that use tobacco products with access to evidence-based smoking cessation supports. Initiation of a smoking cessation program during hospitalization is associated with a decrease in smoking related readmission and a longer post-discharge cessation period.^14,15^ These programs should effectively reach and support patients from communities that are most affected by tobacco use.

| **2.3. Best Practice Standard: Hospital provides buprenorphine treatment for opioid use disorder in the emergency department.** |
| --- |

*Best Practice Standard*. Hospitals should establish guidelines that address the major topics covered by the Massachusetts Hospital Association or equivalent protocols for the administration of buprenorphine in the emergency department.^16^ These guidelines address:

1. A patient assessment protocol
2. Clinical protocol for patients who meet criteria for treatment
3. Expectations for practitioners
4. Discharge plans

*Example*. A number of hospitals, including Johns Hopkins Hospital (MD) and the Massachusetts General Hospital (MA) have access to buprenorphine in the emergency department and have trained clinical providers to administer it.^16,17^

*Background.* Opioid use related drug overdoses resulted in 46,802 deaths in the United States in 2018.^18^ Buprenorphine is a medication that helps people decrease their use of heroin, fentanyl, and other illegal or prescribed opioids – and is associated with a reduction in the risk of death by 50% or more. A randomized controlled trial showed that initiation of buprenorphine in the emergency department was associated with a doubling of the rate of treatment engagement one month later.^19^ On this basis, the American College of Medical Toxicology and other professional associations have endorsed “the administration of buprenorphine in the emergency department as a bridge to long-term addiction treatment.”^20^

| **2.4.  Best Practice Standard: Hospital provides screening, brief intervention, and referral to treatment for alcohol use in the emergency department and hospital.** |
| --- |

*Best Practice Standard*. Hospitals can provide universal screening, subsequent brief intervention, and referral to treatment to patients in the emergency department and hospital as recommended by the ACEP. Hospitals can use the following Substance Abuse and Mental Health Services Administration (SAMHSA) guidelines for SBIRT programs.^21^ These include:

| 1. It is brief (e.g., typically about 5-10 minutes for brief interventions; about 5 to 12 sessions for brief treatments) 2. The screening is universal 3. One or more specific behaviors related to risky alcohol and drug use are targeted 4. It is comprehensive (comprised of screening, brief intervention/treatment, and referral to treatment)   *Substance Abuse and Mental Health Services Administration, 2011*^21^ |
| --- |

*Example.* MedStar Hospital (MD) partnered with the Mosaic Group to implement SBIRT with universal screening in 2016 in the emergency departments of their four hospitals, which was supported by a grant from Behavioral Health Systems Baltimore.^22^ Howard University Hospital (DC) is planning to roll out a program to provide universal SBIRT screening for alcohol and drug use in its hospital, emergency department, and other locations supported by a grant from SAMHSA.^23^

*Background.* Alcohol use significantly contributes to preventable mortality (estimated cause of 255 deaths per day in the US from 2011-2015) and morbidity (for example, use is associated with poor mental health outcomes in children and adolescents).^24,25^ Further, there are racial disparities in the accessibility and quality of alcohol treatment services.^26^ The use of the screening, brief intervention, and referral to treatment (SBIRT) approach in the emergency department for patients with alcohol use disorder has been associated with decreased levels of alcohol use, injury, and return visits to the emergency department.^27^ The American College of Emergency Physicians has endorsed the use of SBIRT models in emergency settings, stating that “emergency medical professionals are positioned and qualified to mitigate the consequences of alcohol abuse through screening programs, brief intervention, and referral to treatment.”^28^

| **2.5. Best Practice Standard: Hospital runs a hospital-based violence prevention program.** |
| --- |

Hospital medical teams can improve outcomes for victims of interpersonal violence by deploying evidence-based violence prevention programs. Hospital-based violence prevention programs can decrease the number of repeat violence-related injuries among victims of violence, increase rates of employment, and increase use of community services.^29,30^

*Best Practice Standard.* Hospitals can implement recommendations made by the National Network of Hospital-Based Violence Intervention Programs for trauma centers and hospitals treating more than 100 assaults per year. Such programs should include:^30^

| 1. On-site crisis intervention specialists to provide a brief crisis intervention 2. Development of a discharge plan 3. Linkage to community-based services including mental health services, mentoring, home visiting, and long-term case management   *National Network of Hospital-Based Violence Intervention Programs*^30^ |
| --- |

*Example*. The Detroit Medical Center Sinai-Grace Hospital (MI) has developed the Detroit Life is Valuable Everyday (DLIVE) program.^31^ This program has violence intervention specialists who engage with survivors of interpersonal violence.

*Background.* Interpersonal violence is bodily or other harm inflicted on an individual by one or more other people, and includes both domestic and community violence.^32^ There were more than 19,000 deaths due to homicide and over 1.6 million non-fatal assault injuries in the United States in 2018.^33,34^ Homicide ranks among the top 5 causes of death for young men ages 15-34 years old in the United States, and in the top 10 causes of nonfatal injury for all individuals ages 10-64.^33,34^ Survivors of interpersonal violence have an increased risk of interpersonal violence victimization in the future.^35^

| **2.6.  Best Practice Standard: Hospital screens for intimate partner violence and refers to services and supports as needed.** |
| --- |

*Best Practice Standard.* Hospitals can implement the approach recommended by the American College of Obstetrics and Gynecology for screening and response to IPV for women of childbearing age per USPSTF recommendations.^36,37^ The approach is stated below:

| 1. Screen for IPV in a private and safe setting with the woman alone and not with her partner, friends, family, or caregiver. 2. Use professional language interpreters and not someone associated with the patient. 3. At the beginning of the assessment, offer a framing statement to show that screening is done universally and not because IPV is suspected. Also, inform patients of the confidentiality of the discussion and exactly what state law mandates that a physician must disclose. 4. Incorporate screening for IPV into the routine medical history by integrating questions into intake forms so that all patients are screened whether or not abuse is suspected. 5. Establish and maintain relationships with community resources for women affected by IPV. 6. Keep printed take-home resource materials such as safety procedures, hotline numbers, and referral information in privately accessible areas such as restrooms and examination rooms. Posters and other educational materials displayed in the office also can be helpful. 7. Ensure that staff receive training about IPV and that training is regularly offered.   *American College of Obstetrics & Gynecology, 2012* ^37^ |
| --- |

*Example*. The Children’s Hospital of Philadelphia (CHOP) as part of its Violence Prevention Initiative universally screens all caregivers of children in the emergency department, and provides staff support and referral resources through a partnership with several community organizations.^38^ Kaiser Permanente Northern California (CA) has a multifaceted approach to IPV, including routine screening and intervention in clinical settings, electronic health record clinical decision support, behavioral health services, and partnership with advocacy organizations.^39^At least 11 Veterans Health Administration medical centers have increased screening and risk assessment for IPV, provision of mental health resources, partnerships with community organizations, and availability of resources to support patients who have experienced IPV.^40^

*Background*. Intimate partner violence, otherwise known as IPV, refers to physical, sexual, and/or psychological harm in an intimate or dating relationship.^37^ Approximately 1 in 3 people living in the United States will experience IPV during their lifetime.^41^ IPV led to at least 2,340 deaths in the US in 2007, 70% of whom were women.^37^ IPV is associated with detrimental health effects, including physical injury, psychological illness, increased rates of female prepartum and intrapartum smoking, and worse pregnancy outcomes.^41,42^ Fewer than half of clinicians routinely report screening for IPV. Screening patients for IPV in the emergency department has been shown to significantly increase detection and provision of appropriate resources, without an increase in IPV as a result of the screening.^43-45^ The American College of Emergency Physicians recommends that “emergency personnel assess patients for IPV, child and elder maltreatment and neglect and emergency physicians are familiar with signs and symptoms of IPV, child and elder maltreatment and neglect.”^46^

| **2.7. Best Practice Standard: Hospital encourages healthy food choices.** |
| --- |

*Best Practice Standard.* Hospitals can implement changes addressing at least five of the eight food categories per the New York City Department of Health and Human services standard.^47^ The categories include Fruits and Vegetables; Sandwiches, Salads, and Entrees; Soups and Sides; Breakfast Breads, Desserts and Snacks; Cooking Method; Beverages; Placement, Promotion and Pricing; and Sustainability.

*Example*. Several hospitals in the New York City area have implemented these standards, including Montefiore Medical Center and Richmond University Medical Center.^47-49^

*Background.* More than 2 in 5 US adults have a body mass index (BMI) that is in the obese category.^50^ People who have obese BMI are at greater risk of developing diabetes, hypertension, cancer, and have an overall higher risk of death.^51-53^ Diets rich in fruits and vegetables and low in trans fat and added sugars are associated with health benefits including lower body weight.^54^ Access to affordable healthy food is also associated with improvements in chronic disease outcomes among people with cardiovascular disease and other conditions.^55,56^ In response to the direct link between food and health, the New York City Department of Health and Human Services developed a set of food standards as part of the Healthy Hospital Food Initiative. A review of this program showed that the hospitals that committed to the program successfully integrated an affordable healthy meal offering, offered more whole grains, offered lower calorie options, and reduced the number of unhealthy items.^57^

| **2.8.Best Practice Standard: Hospital has a social needs screening and referral program.** |
| --- |

*Best Practice Standard*. Hospitals can develop a universal social needs screening and referral program, based on recommendations from the American Hospital Association (AHA) National Academies of Science Engineering and Medicine (NASEM).^58,59^

This program can have two components: screening and referral.

*Screening*. Hospitals can use a validated screening tool such as those published by CMS, HealthLeads, and AAFP, or one of the several developed for children.^60-63^

*Referral*. Hospitals can follow the following AHA recommendations for assisting patients who have a positive social needs screen:^58^

| 1. Define roles and responsibilities of the care team members. Leverage the expertise of other professionals (e.g., social workers, community health workers, community benefit, IT, etc.) within the hospital with knowledge on referrals, community relationships, data, etc. Empower the care team with tools, skills and time to learn, implement and track the screening and referral process. 2. Create a centralized system to capture data and incorporate coding the results of patient screens into an electronic medical record system. 3. Foster partnerships with community-based organizations to create a referral network, raise awareness about services provided and bridge any gaps in care. 4. Develop a referral and community resource database. Integrate the inventory of community resources into a web-based or software system to generate and better track referrals. 5. Close the referral loop by developing a bidirectional process, where the care team follows up with the patient to see if the referral was received and then again in one to two weeks to ensure needs were met. Develop a system to track referrals and measure the success of the screening and referral process.   *American Hospital Association, 2019* ^58^ |
| --- |

*Example*. Boston Medical Center (MA) screens all patients for social needs through the THRIVE Social Determinants of Health Program.^53^ The RWJ Barnabas hospital (NJ) recently rolled out the “Health Beyond the Hospital” program, which will universally screen patients for social needs and refer appropriately.^64^ ProMedica (OH) has also recently expanded its social needs screening to include inpatients in addition to ambulatory patients.^65^

*Background.* The impact of social needs such as food insecurity, transportation, and housing on health is well documented.^66-69^ Social needs screening and connecting patients with resources has been associated with improved health outcomes, yet is not widely done.^70,71^

| **2.9. Best Practice Standard: Hospital offers an infant safe sleep education program.** |
| --- |

*Best Practice Standard.* Hospitals can support healthcare providers and staff working with newborns in the nursery and neonatal intensive care unit (NICU) to “endorse and model” safe sleep practices per the recommendations of the American Academy of Pediatrics. These recommendations include the following: ^72^

| 1. In the NICU: Modelling and implementing all sudden infant death syndrome (SIDS) risk-reduction recommendations as soon as the infant is medically stable and well before anticipated discharge. 2. In the Newborn Nursery: Modelling and implementing all SIDS risk-reduction recommendations beginning at birth. 3. All physicians, nurses, and other health care providers working on mother-baby units should receive education on safe infant sleep. Health care providers should screen for and recommend safe sleep practices at each visit for infants up to 1 year old. Families who do not have a safe sleep space for their infant should be provided with information about low-cost or free cribs or play yards. 4. Ensure that hospital policies are consistent with updated safe sleep recommendations and that infant sleep spaces (bassinets, cribs) meet safe sleep standards. 5. Public education should continue for all who care for infants, including parents, child care providers, grandparents, foster parents, and babysitters, and should include strategies for overcoming barriers to behavior change.   American Academy of Pediatrics, 2016 ^72^ |
| --- |

*Example*. In the Georgia Safe to Sleep Hospital Initiative (GA), hospitals provide safe sleep education for caregivers while the baby is still in the hospital, information on safe sleep practices, and travel bassinets to low-income families.^73^ The initiative was found to increase the rate of safe sleep practices, including decreasing the practice of bed sharing.

*Background*. Every year in the United States, approximately 3,500 infant deaths occur as a result of unsafe sleep practices.^74^ Hospitals can play a critical role in teaching, modeling, and reinforcing safe sleep practices with caregivers of neonates and infants. The CDC developed the “Back to Sleep” guidelines for safe infant sleep in 1994. A subsequent study (1999) showed an increase in appropriate sleep practices in the 13 states studied.^75^

| **2.10.  Best Practice Standard: Hospital adopts 10 practices to support breastfeeding.** |
| --- |

*Best Practice Standard.* Hospitals can follow all 10 of the “baby friendly” standards supported by the World Health Organization to support and promote breastfeeding.^76^

| 1. Facility breastfeeding policy (following the International code of marketing of Breast-milk substitutes, breastfeeding policy that is shared with parents, data monitoring of rates of breastfeeding) 2. Staff training in breastfeeding support 3. Clinical discussion of breastfeeding before birth 4. After birth skin to skin contact and initiation of breastfeeding as soon as clinically possible 5. Continued support of mothers to breastfeed 6. Only providing supplemental infant formula when clinically necessary 7. Allowing mothers to stay in the same room as the baby (including in the NICU if physically and logistically feasible) 8. Providing teaching for mothers to identify baby feeding cues 9. Providing instruction on use and risks of non-breast objects into the baby’s mouth (such as pacifiers) 10. Coordination of follow up outpatient appointment at time of hospital discharge   *World Health Organization, 2018* ^76^ |
| --- |

*Example*. Boston Medical Center (MA) successfully implemented these policies and substantially increased initiation of breastfeeding among new mothers.^77^

*Background*. Breastfeeding is associated with a multitude of benefits to newborns at birth and later in life, as well as to the mother, including lower rates of neonatal and infant mortality, decreased rates of childhood obesity, and lower risk of breast cancer in the mother.^78-80^ Hospitals are in a unique position to assist mothers and babies with initiation of feeding patterns to optimize health outcomes. The Baby Friendly Hospital Initiative was developed in 1991 to help hospitals increase breastfeeding rates. Each of the ten components of the Baby Friendly designation is evidence based.^81^ Further, full implementation of these recommendations has been associated with an increase in short, medium, and long-term breastfeeding rates.^82^

| **2.11.  Best Practice Standard: Hospital offers contraception treatment and counselling to patients immediately postpartum.** |
| --- |

*Best Practice Standard*. Hospitals can offer contraceptive treatment and counseling, including LARC, after birth as part of programs that are consistent with the recommendations by the American College of Obstetricians and Gynecologists.^83^ These recommendations highlight the importance of prenatal and postnatal counselling and discussions about the risks and benefits of LARC, as well as the option for receiving LARC placement at the postpartum medical appointment.

*Example*. Michigan Medicine (MI) offers LARC to patients after giving birth in the inpatient setting.^84^ This was made possible by a change to Medicaid rules allowing for reimbursement of providing long acting reversible contraception to inpatients.

*Background*. Becoming pregnant less than one year after giving birth can have negative health consequences for the pregnant individual and baby, including pre-term delivery, low birth weight, gestational diabetes, and elevated infant and maternal mortality.^85-88^ Initiation of long acting reversible contraception (LARC) immediately after giving birth to patients who do not want to become pregnant again is safe and convenient and reduces the risk of a short interpregnancy interval.^89^ Given the history of reproductive rights coercion and control, particularly towards Black and Native American women, incarcerated women, and women of low socioeconomic status, it is essential that LARC programs understand and support each patient’s health autonomy and reproductive goals. This includes providing comprehensive contraceptive counseling prior to birth.

| **2.12. Best Practice Standard: Hospital implements practices to reduce falls and optimize mobility for elderly patients per the Age Friendly Hospital Program.** |
| --- |

*Best Practice Standard*. Hospitals can implement the following Age Friendly Health System practices established by the Institute for Healthcare Improvement (IHI) to reduce falls and optimize mobility:

| 1. Screen for mobility limitations and document the results 2. Ensure early, frequent, and safe mobility   *Institute for Healthcare Improvement, 2019* ^90^ |
| --- |

*Example*. The Institute for Healthcare Improvement keeps a comprehensive list of health systems recognized as age-friendly.^91^ Five “pilot health systems” including Anne Arundel Medical Center (MD), Ascension (MO)​, Kaiser Permanente (CA), Providence St. Joseph Health (WA), and Trinity Health (MI) have implemented age-friendly practices in their hospitals.

*Background.* Falls are the leading cause of preventable injury for older adults.^92^ Recent hospitalization is associated with a higher prevalence of falls, while improved functional status is correlated with increased life expectancy.^93,94^ The Age Friendly Health System is an evidence-based set of practices to improve health outcomes and care for the elderly. Several of these practices focus on reducing the risk of falls after discharge.^90^

| **3.1. Best Practice Standard: Hospital performs a community needs assessment in collaboration with the department of health, community based organizations, and community residents.** |
| --- |

*Best Practice Standard*. Hospitals can follow the standard set by the American Public Health Association (APHA) and collaborators stating that “hospitals should consult with both the state health department where the facility is licensed and the local health department where the facility is located.”^95^ Hospitals should also “seek input from community representatives” to develop the assessment and plan.

*Example*. The Health Planning Council of Northeast Florida (FL) is an example of a collaboration between several hospitals (the Mayo Clinic, Shands Jacksonville Medical Center, St. Vincent’s HealthCare and others) and health departments (including the Clay County Health Department, Duval County Health Department, and Putnam County Health Department).^96^ These groups worked together to compile clinical and community data to develop a community health needs assessment for the area. A similar approach was taken by the Health Collaborative in Cincinnati, which included 20 hospitals, the Cincinnati Health Department, the Hamilton County Public Health Department, and others.^97^

*Background*. As part of tax reporting requirements established by the Affordable Care Act, all non-profit hospitals are required to conduct a community health needs assessment every three years. Collaboration with local health departments and consultant-led community health needs assessments is associated with higher-quality assessments.^98^

| **3.2. Best Practice Standard: Hospital provides meaningful support for a community based hypertension control program.** |
| --- |

*Best Practice Standard*. Hospitals can provide meaningful support for community-based hypertension control programs at barbershops, beauty salons, and religious institutions based on established evidence-based models.

Recognizing the uniqueness of each community and hospital, what constitutes a meaningful contribution should be determined by each hospital in conjunction with their community partners. At a minimum, we propose defining a meaningful contribution to include 1) hospital involvement at least 12 months; 2) significant contributions of personnel and staff time, clinical coordination, or financial investments and 3) a community partner who will endorse the importance of the hospital’s work in the area for community health.

*Examples*. Cedar Sinai (TX) has a program involving local barbershops in for blood pressure screenings, health promotion, and referrals to care.^99^ The program in Texas showed that a significantly higher percentage of people in the intervention barber shops had controlled blood pressure. A similar program is being piloted at Vanderbilt Medical Center (TN).^100^

*Background*. Hypertension affects almost 50% of the American adult population, and African Americans have earlier onset and more severe hypertension compared to whites.^101,102^ Given the high number of patients impacted by hypertension, and given that it is largely treatable, hospitals have an opportunity to have high impact interventions. Further, hypertension is largely a chronic illness, and stems from many modifiable risk factors. Several successful hypertension-related interventions have grown out of community and healthcare institution collaborations. Evidence-based programs include community led hypertension screening programs at barbershops, beauty salons, and churches, and multifactorial community hypertension health education initiatives.^103-106^ The American Heart Association Hypertension Practice Guidelines call for “population-based initiatives … to reduce the global burden of raised blood pressure.”^107^

| **3.3. Best Practice Standard: Hospital provides meaningful support for a community based diabetes prevention program.** |
| --- |

*Best Practice Standard*. Hospitals can provide meaningful support for implementation of a community-based diabetes prevention program.

Recognizing the uniqueness of each community and hospital, what constitutes a meaningful contribution should be determined by each hospital in conjunction with their community partners. At a minimum, we propose defining a meaningful contribution to include 1) hospital involvement at least 12 months; 2) significant contributions of personnel and staff time, clinical coordination, or financial investments and 3) a community partner who will endorse the importance of the hospital’s work in the area for community health.

*Examples*. The 11 hospitals of the Montefiore Health System (NY) integrated the Diabetes Prevention Program into its health system through a partnership with the Young Men's Christian Association of Greater New York.^108^ Several hospitals in West Virginia partnered with the state health department to implement the National Diabetes Prevention Program for employees of surrounding communities, demonstrating significant return on investment and incentivizing hospitals to continue these programs.^109^

*Background*. One in ten children and adults in the United States have type 2 diabetes, a number that has continued to increase over time. Diabetes contributes significantly to morbidity and mortality, increases the risk of cardiovascular disease, and is the seventh leading cause of death in the United States.^108^ Diet and activity modification programs such as those in the Diabetes Prevention Program trial have been shown to decrease the incidence of types 2 diabetes.^110^ Participants with impaired glucose tolerance in the Program had over a 50% decrease in the incidence of diabetes within 2.8 years of the intervention. The Center for Disease Control and Prevention (CDC) has developed a diabetes prevention curriculum from this intervention, which has been implemented in many communities through partnerships with nonprofit groups.^111^

| **3.4. Best Practice Standard: Hospital provides meaningful support for an evidence-based home visiting program.** |
| --- |

*Best Practice Standard*. Hospitals can provide meaningful support for an evidence-based home visiting program such as the Nurse Partnership program referenced below.

Recognizing the uniqueness of each community and hospital, what constitutes a meaningful contribution should be determined by each hospital in conjunction with their community partners. At a minimum, we propose defining a meaningful contribution to include 1) hospital involvement at least 12 months; 2) significant contributions of personnel and staff time, clinical coordination, or financial investments; and 3) a community partner who will endorse the importance of the hospital’s work in the area for community health.

*Example*. The Welcome Baby program through the Maternal and Child Health Access partnership with California Hospital Medical Center (CA) provides home visit services to new mothers, beginning when the baby is first born in the hospital. Since its inception in 2009, the program has been expanded to 14 hospitals.^112^

*Background*. Home nursing visit programs for new mothers and their babies have been shown to result in many positive health outcomes including decreased prenatal maternal smoking, improved child cognitive performance, and reduced child abuse and neglect.^113^

The Nurse Partnership program in Memphis (TN) found that the participants in the program had lower risk of hypertension during pregnancy, as well as lower mortality, suicide, drug overdose, and homicide rates.^114^ Additionally, babies in the program had lower rates of preventable mortality, homicide, and hospitalization during their first 2 years of life. The American Academy of Pediatrics recommends that health systems “ensure that all home-visiting programs incorporate evidence-based strategies and achieve program fidelity to ensure effectiveness.”^115^

| **3.5. Best Practice Standard: Hospital provides meaningful support for training and work of community health workers*.*** |
| --- |

*Best Practice Standard*. Hospitals can provide meaningful support to community health worker programs in the context of value-based care initiatives and in coordination with primary care services in the community. This standard can be met by the hospital itself hiring and training community health workers or by making meaningful contributions to programs in the community.

Recognizing the uniqueness of each community and hospital, what constitutes a meaningful contribution should be determined by each hospital in conjunction with their community partners. At a minimum, we propose defining a meaningful contribution to include 1) hospital involvement at least 12 months; 2) significant contributions of personnel and staff time, clinical coordination, or financial investments and 3) a community partner who will endorse the importance of the hospital’s work in the area for community health.

*Example*. The Greenville Health System (SC) PASOs program connects community members with community health workers, social workers, and healthcare providers, and is aimed at individuals in the Latinx community.^116^ Penn Medicine (PA) has provided financial support for the development of the IMPaCT program at the Penn Center, which has allowed community health workers to engage with thousands of patients in Philadelphia.^117,118^

*Background*. Community health workers are “frontline public health workers who are trusted members of and/or have an unusually close understanding of the community served.”^119^ While some specifics of local definitions may vary, community health workers generally are from the community that they serve, and share the attributes and life experiences of their clients. These individuals are trained to work with patients at home and in their places of work, study and worship to further health through education, support, and connecting patients to resources. Community health workers can engage with patients via multiple communication modalities including telephone contact and home visits.

There is substantial evidence showing that community health workers improve outcomes for patients with diabetes and hypertension, among other conditions.^120^ Assistance of recently hospitalized patients by community health workers may reduce rates of readmission and improve patients’ ability to effectively engage with preventative care.^121,122^ The American Hospital Association recommends that “hospitals and health systems can incorporate community health workers into their workforce, extending care beyond the hospital or clinic walls to help bridge gaps in care, expand access to care and, ultimately, improve health outcomes for high-risk patients.”^123^

| **3.6. Best Practice Standard: Hospital makes meaningful contributions to supporting school success.** |
| --- |

*Best Practice Standard.* Hospitals can make meaningful contributions to supporting school success through one or more of three types of activities:

| 1. Direct services, such as comprehensive school health services. 2. Collaborative work with education system officials to improve outcomes such as third grade reading or absenteeism. 3. Strategic partnerships with schools to provide training and employment for graduates. |
| --- |

Recognizing the uniqueness of each community and hospital, what constitutes a meaningful contribution should be determined by each hospital in conjunction with their community partners. At a minimum, we propose defining a meaningful contribution to include 1) hospital involvement at least 12 months; 2) significant contributions of personnel and staff time, clinical coordination, or financial investments; and 3) a community partner who will endorse the importance of the hospital’s work in the area for community health.

*Example.* The Akron Children’s Hospital (OH) developed a partnership with the local school system to provide more in-school healthcare services. ^124^ The Dell Children’s Hospital (TX) provided financial assistance to the Austin Independent School District when it was facing a budget shortfall that could potentially necessitate laying off school nurses.^124^ Cincinnati Children's Hospital (OH) also invests in a variety of initiatives to improve reading skills for children in the Cincinnati area.^125^

*Background*. Education and health are intrinsically linked. A 25 year old with a college degree can expect to live 10 years longer than their 25 year old counterpart without a high school degree.^105^ Chronic absenteeism is associated with risky behavioral choices and poor school performance.^126^ A low literacy level is associated with worse health literacy and health outcomes.^127,128^ Yet, across the United States there are great disparities in access to elementary and higher education. The American Academy of Pediatrics notes that “pediatricians and their colleagues caring for children in the medical setting have opportunities at the individual patient and/or family, practice, and population levels to promote school attendance and reduce chronic absenteeism and resulting health disparities.”^126^

| **3.7. Best Practice Standard: Hospital meaningfully supports expanding access to fresh, affordable, healthy foods in the community.** |
| --- |

*Best Practice Standard.* Hospitals can make meaningful investments to increase access to affordable healthy food in their communities consistent with recommendations from the Commission to Build a Healthier America through one or more of the following activities or through a hospital food pantry that offers healthy food.^129^

| 1. Investing in development of grocery stores in low food access neighborhoods 2. Providing financial support to community groups working on food access 3. Providing on-campus farmers markets with a majority of affordable items, acceptance of food benefits, and advertising to the surrounding community   *^-^Commission to Build a Healthier America*^129^ |
| --- |

Recognizing the uniqueness of each community and hospital, what constitutes a meaningful contribution should be determined by each hospital in conjunction with their community partners. At a minimum, we propose defining a meaningful contribution to include 1) hospital involvement at least 12 months; 2) significant contributions of personnel and staff time, clinical coordination, or financial investments; and 3) a community partner who will endorse the importance of the hospital’s work in the area for community health.

*Example.* Kaiser Permanente supports a network of farmers markets at their clinical locations and in the community.^130^ Another example is the St. Mary Medical Center Farm to Families Initiative (PA) which provides affordable, healthy food to 1,000 families.^131^ The Medical Center also developed Fresh Connect, a healthy food delivery service and mobile open market to individuals in need of these services.

*Background*. In 2019 over 10% of US families faced food insecurity, which is the lack of enough food to meet basic nutritional and health needs.^132^ Socioeconomically disadvantaged, rural, and racially segregated Black and Brown communities are disproportionately impacted by limited access to healthy, nutritious, and affordable food, geographic areas also referred to as “food deserts.”^133,134^ Both adults and children that are impacted by food insecurity have higher rates of iron deficiency anemia, tooth decay and depression.^66^ Adults experiencing food insecurity also have higher risk of cardiovascular disease and related hospitalization, diabetes and limitations to activities of daily living, and children experiencing food insecurity have higher rates of asthma.^66^ Improving healthy food availability in communities is associated with behaviors that are linked to chronic disease management for conditions like diabetes and cardiovascular disease.^135^ The Robert Wood Johnson Foundation’s Commission to Build a Healthier America has recommended that the health care system “create public-private partnerships to open and manage full-service grocery stores in communities without access to healthful food.”^135^

| **3.8. Best Practice Standard: Hospital invests in expanding or improving healthy, affordable housing in the community.** |
| --- |

*Best Practice Standard.* Hospitals and health systems can make meaningful investments to increase access to safe, affordable housing in safe and economically vibrant communities. Importantly, hospitals should be vigilant that their efforts do not contribute to further gentrification and dislocation of vulnerable populations. To meet this standard, hospitals can make meaningful contributions to one or more of the following, based on successful examples of these efforts by hospitals:

1. Creating and sustaining community partnerships to improve economic and housing stability
2. Conducting home safety and environmental hazards assessments, repairs, and renovations to improve housing stability, housing quality, and health
3. Developing affordable housing units for disabled, elderly or individuals experiencing homelessness and families and low-income families with children in their catchment area
4. Partnering with public housing authorities, nonprofit affordable housing developers, and others to increase access and availability of safe, affordable housing in safe, economically vibrant communities within their catchment area
5. Providing or supporting co-location of health services to meet the needs of chronically ill and high-needs populations in settings such as transitional housing and permanent supportive housing

*Example*. The Bon Secours Baltimore Health System (MD) has invested in affordable housing units in the community.^136^ Children's Mercy Kansas City's Healthy Homes program provides assessments of home safety and environment.^137^ Nationwide Children’s Hospital’s (KS) “Healthy Homes” program provides funding for renovation of affordable existing and new rental properties and homes.^138^ The initiative is funded through the Center for Community Investment grant program.^139^ Through the Better Health Through Housing program, the University of Illinois Hospital (IL) partners with the Chicago Center for Housing and Health to provide social and economic supports to connect patients with housing.^140^ The New Jersey Housing and Mortgage Finance Agency has a supportive housing partnership with local hospitals, including St. Joseph’s Health (NJ).^141^

*Background*. The United States is experiencing a growing housing crisis. Since the great recession, the proportion of United States residents facing “worst case” housing needs has increased. People with “worst case” housing needs are defined as those who do not receive housing assistance from the government, pay over half of income towards housing, and have inadequate living conditions.^142^ Lack of access to affordable, safe housing contributes to poor health including increased risk of respiratory illness, toxic exposures, falls, and premature death.^143,144^ Metrics that assess the housing challenges in a community include the Index of Isolation, Index of Dissimilarity, Index of Neighborhood Disinvestment, and Heat Vulnerability Index.^145-148^ When housing costs consume a disproportionate share of household financial resources, individuals and families may forego other necessities such as purchasing food or medications.^149,150^ The American Hospital Association recommends that “hospitals and health systems implement strategies and programs to improve housing stability.” Investments in housing fall into three categories:

1. Financial support for expanding high quality, affordable housing^136^
2. Financial support for assessment and renovation of existing affordable housing^151^
3. Partnership with state-led finance housing agencies, local community development financial institutions, or other community agencies to increase the supply of safe, affordable housing through redevelopment or revitalization initiatives.^152,153^

| **4.1. Best Practice Standard: Hospital has a five-year plan for achieving diversity in board and top management and measures progress towards this goal.** |
| --- |

*Best Practice Standard*. Hospitals can follow the recommendations of the NAACP to “develop a measurable and achievable goal for diversity in...top management … and a five-year plan to accomplish it.”^154^ Hospitals should also track progress towards this goal. The definition of diversity encompasses not only race and gender but other factors such as sexual orientation, age, ability, and income. Key components of such plans may include one or more of the following strategies:^155^

| 1. Voluntary diversity targets 2. Selecting a diverse candidate pool 3. Providing mentorship to board candidates 4. Consideration of non-CEO, non “traditional pipeline” candidates 5. Term and age limits 6. Larger board size 7. Board performance evaluation   *U.S. Government Accountability Office* ^155^ |
| --- |

*Example.* Christus Health (TX) has worked to increase racial, ethnic, gender and age diversity in its leadership in several ways, including making leadership board diversity and inclusion goals in its organizational plan, monitoring diversity of the board, and having a requirement that at least 33% of the candidates in a board member hiring pool are “diverse.” ^156^These efforts among others have resulted in an increase in the percent of trustees identified as racially diverse (from 10 to 35%) and as women (from 10 to 40%). Equally importantly, a member of the board reported that the increased diversity of the board has led to a “greater focus on community needs.” The Robert Wood Johnson University Hospital implemented several programs to support hiring and retention of underrepresented minorities in leadership positions, including a “formal diversity and inclusion program,” collection and review of diversity statistics, mentorship and pipeline programs, and supportive working groups.^157^ This work led to an increase from 17% (2011) to 22% (2015) of minorities on the hospital board, as well as an increase in the percentage of minorities in leadership from 4% (2012) to 32% (2015).

*Background*. Historically marginalized groups, such as racial minorities and women, have significantly lower representation in hospital leadership, and disproportionately perform lower paying jobs in the United States healthcare industry.^158,159^ Reasons include institutional factors such as biased recruitment, hiring, and promotion practices. Many hospital systems have recognized the urgent need to implement systematic changes to increase diversity in all levels of hospital leadership.^160^ In addition to the benefits inherent in hiring talented professionals and working towards institutional equity, hospital leaders who reflect the diversity of the community may be better positioned to “engender greater implementation of initiatives to reduce disparities and promote health equity.” ^161^

| **4.2. Best Practice Standard: Hospital pays all employees a minimum hourly rate based on the local living wage.** |
| --- |

*Best Practice Standard.* Hospitals can establish a minimum wage based on the MIT living wage calculator set to “single adult with no children.” This wage should be close to or exceeding $15 per hour. This wage should apply to all hospital employees, including contractors, such as environmental staff.

*Example*. The University of Arkansas for Medical Sciences (AR) established a minimum wage of $14.50 per hour for all employees of the institution in 2018. This figure was based on the living wage in its home county of Pulaski County, Arkansas.

*Background*. A living wage is defined as the amount of money that a full-time working person needs to earn to cover basic living expenses and health needs of themselves and their dependents.^162^ The living wage is generally higher than the federal minimum wage of $7.25 per hour.^163^ Every one dollar increase in earnings above the federal minimum wage is associated with a decrease in the number of infants with a low birth weight, postneonatal mortality, and smoking rate.^164,165^ Implementing a minimum wage of $15 per hour would reduce the poverty rate of female health workers by 27.1%.^166^ The Institute for Healthcare Improvement has concluded that “a living wage for healthcare workers is essential to achieve health equity.”  ^167^

| **4.3. Best Practice Standard: Hospital has a minority owned business purchasing and procurement goal and measures progress towards this goal.** |
| --- |

*Best Practice Standard.* Hospitals can make minority owned business purchasing and procurement goals and measure their progress towards these goals. This standard is based on the NAACP recommendation that “healthcare firms can begin tracking supplier diversity in greater detail and ask primary suppliers to include a specific level of diversity in their subcontracted work as well.”  ^154^

*Example*. Several hospitals including the University of Pittsburgh Medical Center (PA), the Mayo Clinic (MN), and Johns Hopkins Hospital (MD) have minority owned business procurement policies and goals.^168-170^ The Kaiser Hospital System has a policy that also requires that suppliers provide certification that they have diverse ownership including “minority, woman, LGBT, veteran, veteran-disabled, and disabled” individuals.^171^

*Background*. Income inequality has a deleterious effect on health outcomes, especially for those at the lower end of the income spectrum.^172^ Historically, minority communities have had less opportunity for economic development and investment due to governmental loan practices and systemic racism. Hospitals have enormous purchasing power, and thus have an opportunity to contribute to equitable economic opportunities by intentionally investing in minority owned businesses.^173,174^ Life expectancy is closely tied to income.^175^ Further, as income disparities are particularly stark along racial lines, income inequality contributes to worse health disparities.^176,177^

| **4.4. Best Practice Standard: Hospital supports access to affordable high-quality child care for children of all full and part-time employees.** |
| --- |

*Best Practice Standard*. Hospitals can provide support for all full- and part-time employees to access quality, affordable child care. Hospitals can meet this standard in one of several ways, per the recommendations by the International Finance Group (IFG):^178^

| 1. On-site childcare centers (run by the employer or a third party) 2. Support for off- or near-site childcare centers 3. Childcare vouchers, subsidies, stipends, discounts, and other supported access to services   International Finance Group, 2019^178^ |
| --- |

*Example*. University Hospital Parma (OH) provides early childhood education and care to children of employees and physicians, and recently expanded the service to children of people not employed by the hospital.^179^ Stanford Hospital offers two part-time nursery schools, as well as two full-time child care programs to all of its employees.^180^

*Background*. Enrollment in quality early childcare is associated with better behavioral and cognitive outcomes for children.^181^ Additionally, women make up 76% of the healthcare workforce, yet lack of childcare is associated with decreased participation of women in the workforce.^182,183^ In most western societies women have typically been charged with the financially uncompensated job of raising children, thus, having access to childcare improves economic opportunities for women. The American Academy of Pediatrics policy emphasizes the benefit of universal early childhood programs. Beyond basic child care services, there are a myriad of health and learning benefits for children who participate in programs with an education component.^184-186^

| **4.5. Best Practice Standard: Hospital provides paid sick leave to all employees.** |
| --- |

*Best Practice Standard*. Hospitals and their contractors can provide universal paid sick leave to their employees, including ensuring access to sick leave for contract workers and non-clinical staff such as food service employees and environmental cleaning staff. Hospitals should also establish mechanisms to ensure that utilizing sick leave policies does not result in negative repercussions for employees. Hospitals can use the policy adopted by the federal government for covered employers during the COVID-19 pandemic as a framework for their own practices:  ^187^

| 1. Two weeks (up to 80 hours) of paid sick leave at the employee’s regular rate of pay where the employee is unable to work 2. Two weeks (up to 80 hours) of paid sick leave at two-thirds the employee’s regular rate of pay because the employee is unable to work because of a bona fide need to care for others 3. Up to an additional 10 weeks of paid expanded family and medical leave at two-thirds the employee’s regular rate of pay   *United States Department of Labor, 2020* ^187^ |
| --- |

*Example*. University of Rochester Medical Center (NY) provides 10 work days of paid sick leave to all employees yearly.^188,189^ Barnes Jewish Hospital (MO) provides 23 days of paid time off to employees which can be used for vacation, sick leave, or other purposes.^190^

*Background*. Paid sick leave has both immediate and long term health benefits. Individuals with an acute illness are more likely to stay home if they have paid sick leave, which decreases the spread of contagious illnesses to others.^191^ Further, employees with paid sick leave are more likely to have preventative health care (pap test, mammography, endoscopy, flu vaccine) and see a doctor compared to those without paid sick leave.^192,193^ Employees without paid sick leave are also more likely to delay seeking medical care for themselves and for family members such as their children.^191,194^ The American Medical Association (AMA) “supports employer policies that allow employees to accrue paid time off and to use such time to care for themselves or a family member.” ^195^ In addition to the AMA, the National Association of County and City Health Officials (NACCHO) supports paid sick leave policy based on its positive impacts on public health.^196^

| **4.6. Best Practice Standard: Hospital adopts a “do no harm” collections policy.** |
| --- |

*Best Practice Standard.* Hospitals can adopt four recommendations made by the nonprofit organization Community Catalyst for Methodist Hospital in Memphis to assure that their billing practices are reasonable and one from the American Hospital Association on access to information about financial assistance.^197^ These include the following:

| 1. Base eligibility for financial help on need, not insurance status^1^ 2. Adopt a Do-No-Harm collections policy, which prioritizes establishing reasonable payment plans over legal actions^1^ 3. Stop wage garnishments^1^ 4. Provide training to hospital staff about financial aid policies and legal requirements^1^ 5. Hospitals should communicate [financial assistance] information to patients in a way that is easy to understand, culturally appropriate and in the most prevalent languages used in their communities.^2^   ^1^*Community Catalyst, 2019* ^197^  *^2^American Hospital Association* ^198^ |
| --- |

*Example.* The St. Luke’s University Health Network (PA) provides a financial assistance program that includes patients with insurance.^199^

*Background*. An article from the New York Times in 2019 reported that “hospitals across the country are increasingly suing patients for unpaid bills, a step many institutions were long unwilling to take.” ^200^ Medical debt is associated with delays in medical care and treatment.^201^ Further, among individuals who experience homelessness or have less than a 12th grade education, debt collection is associated with a decrease in healthcare utilization.^202^ Importantly, medical debt can affect patients regardless of their insurance status. The American Association of Medical Colleges has stated, “The mission of every hospital in America is to serve the health care needs of people in its community – part of that commitment includes treating patients with dignity and respect from the bedside to the billing office.” ^203^

| **4.7. Best Practice Standard: Hospital has a returning citizens employment program.** |
| --- |

*Best Practice Standard*. Hospitals can take the following steps toward easing restrictions on the hiring of people with criminal records recommended by the NAACP and its partners:^204^

| 1. Meet with other community organizations and employers regarding inclusive hiring policies and the recommendations in this proposal. 2. Review and amend hiring policies to lift the potential “blanket ban” against hiring those convicted of felonies and/or drug offenses. 3. Review and amend existing policies to lift the automatic termination of employment for associates arrested and convicted of non-work-related felonies and/or drug offenses. 4. Develop formal partnerships with intermediaries to identify and prepare potential candidates for employment. Also, employers may consider the development of regional or local plans to partner with available state, municipal, or nonprofit intermediaries currently working in various areas. 5. Develop a procedure to consider “evidence of rehabilitation” and to grant a “waiver” to allow for the hire of applicants with criminal records when they show evidence of rehabilitation, treatment, and/or demonstrate a connection with a support network (e.g., an intermediary). 6. Work with the NAACP or other community partners to engage in a series of research and program activities designed to monitor the successes and challenges associated with the program.   *NAACP*  ^204^ |
| --- |

*Example*. Sinai Health System (IL) partnered with the Safer Foundation and developed the Sinai Pathway Program, which pairs an “employment referral pipeline with a wrap around support system” for returning citizens.^205^ Johns Hopkins Hospital (MD), the Henry Ford Health System (MI), MetroHealth (OH), and University Hospitals (OH) also have returning citizen employment programs.^205,206^

*Background.* The National Employment Law Project estimates that 70 million adults in the United States have an arrest or conviction record.^207^ People with criminal records – especially people of color – are unemployed at higher rates than the general population, limiting their opportunities to support their own health as well as the health of their families and communities.^208^ The development of returning citizen employment programs for individuals who were recently incarcerated is an effective way of connecting people in this situation with employment. The NAACP has a set of best practice guidelines about actions employers can take to provide employment programs that are inclusive of returning citizens. These recommendations include removing complete bans on hiring individuals with a prior felony conviction, removing automatic termination of people convicted of felonies, and developing partnerships with other organizations to support returning citizens' return to the workplace.^204^

| **4.8. Best Practice Standard: Hospital publishes plans for advancing sustainability.** |
| --- |

*Best Practice Standard*. Hospitals can develop and publish their sustainability statement, baseline statistics (energy use, water consumption, and waste stream), and plans to improve. The plans should address at least one sustainability opportunity area: energy, water, supply chain, waste, and commissioning/retrocommissioning. Hospitals and health systems can follow all six steps of the American Hospital Association (AHA) model for improving sustainability as listed below as they develop these plans.^209^

| 1. Make the commitment 2. Create a culture for supporting environmental sustainability 3. Support and finance environmental sustainability 4. Set goals and measure, report and evaluate change 5. Celebrate and share successes 6. Continue to assess and identify new opportunities   *American Hospital Association, 2014* ^209^ |
| --- |

*Example*. Several hospitals have used the AHA model to improve their sustainability. Memorial Hermann Health System (TX) invested in retrocommissioning among other activities to improve energy efficiency.^210^ Wentworth-Douglass Hospital (NH) identified energy and water saving opportunities to decrease operating costs.^211^ Carilion New River Valley Medical Center (VA) benchmarked and monitored energy use to identify opportunities for efficiency improvements.^212^ Sullivan County Community Hospital (IN) hired a utility consultant to identify ways to improve efficiency with existing equipment.^213^

*Background.* The CDC has stated that “climate change... influences human health and disease in numerous ways.” ^214^ Indeed, climate change is a public health issue, and is expected to disproportionately impact people with lower socioeconomic status, further reinforcing social inequalities.^215,216^ In addition to climate change, air and water pollution have also been shown to negatively affect population health, with socioeconomically disadvantaged populations bearing a disproportionate share of these consequences.^217-219^ Many businesses have already followed the call to develop better sustainable practices, including following the goals of the Paris Climate Accord or becoming LEED certified.^220,221^

1. McCaffree MA. AMA policy on smoke-free environments and workplaces.

2. Wang TW. Smoke-free and tobacco-free policies in colleges and universities ― united states and territories, 2017. *MMWR Morb Mortal Wkly Rep*. 2018;67. https://www.cdc.gov/mmwr/volumes/67/wr/mm6724a4.htm. Accessed Jan 1, 2022. doi: 10.15585/mmwr.mm6724a4.

3. Tobacco Free Kids. Electronic cigarettes should be included in smoke-free laws. https://www.tobaccofreekids.org/assets/factsheets/0387.pdf.

4. Veterans Health Administration. Smoke-free VA health care facilities - veterans health administration. https://www.va.gov/health/smokefree/. Accessed Jan 1, 2022.

5. UW hospital smoke and tobacco free policy. https://www.uwhealth.org/patient-guides/uw-hospital/smoke-and-tobacco-free-policy/12798.

6. Tobacco control interventions. https://www.cdc.gov/policy/hst/hi5/tobaccointerventions/index.html Web site. https://www.cdc.gov/policy/hst/hi5/tobaccointerventions/index.html. Accessed Jan 1, 2022.

7. United States Public Health Service Office of the Surgeon General National Center for Chronic Disease Prevention and Health Promotion (US) Office on Smoking and Health. Clinical-, system-, and population-level strategies that promote smoking cessation. In: *Smoking cessation: A report of the surgeon general.* . https://www.ncbi.nlm.nih.gov/books/NBK555600/.

8. Inpatient tobacco-cessation program about to begin at UW hospital. UW-CTRI Web site. https://ctri.wisc.edu/2018/05/14/inpatient-tobacco-cessation-program-about-to-begin-at-uw-hospital/. Updated 2018. Accessed Jan 1, 2022.

9. Smoking & tobacco use fast facts. Centers for Disease Control and Prevention Web site. https://www.cdc.gov/tobacco/data_statistics/fact_sheets/fast_facts/index.htm. Updated 2021. Accessed Jan 1, 2022.

10. Jemal A, Thun MJ, Ries LAG, et al. Annual report to the nation on the status of cancer, 1975–2005, featuring trends in lung cancer, tobacco use, and tobacco control. *JNCI: Journal of the National Cancer Institute*. 2008;100(23):1672-1694. https://doi.org/10.1093/jnci/djn389. Accessed Jan 1, 2022. doi: 10.1093/jnci/djn389.

11. Rigotti NA, Clair C. Managing tobacco use: The neglected cardiovascular disease risk factor. *European Heart Journal*. 2013;34(42):3259-3267. https://doi.org/10.1093/eurheartj/eht352. Accessed Jan 1, 2022. doi: 10.1093/eurheartj/eht352.

12. Virdis A, Giannarelli C, Fritsch Neves M, Taddei S, Ghiadoni L. Cigarette smoking and hypertension. *Current Pharmaceutical Design*. 2010;16(23):2518-2525. https://www.ingentaconnect.com/content/ben/cpd/2010/00000016/00000023/art00003. Accessed Jan 1, 2022.

13. Hanrahan JP, Tager IB, Segal MR, et al. The effect of maternal smoking during pregnancy on early infant lung function. *Am Rev Respir Dis*. 1992;145(5):1129-1135. https://www.atsjournals.org/doi/abs/10.1164/ajrccm/145.5.1129. Accessed Jan 1, 2022. doi: 10.1164/ajrccm/145.5.1129.

14. Reid RD, Mullen K, Slovinec D'Angelo ME, et al. Smoking cessation for hospitalized smokers: An evaluation of the “Ottawa model”. *Nicotine Tob Res*. 2010;12(1):11-18. https://academic.oup.com/ntr/article/12/1/11/998651. Accessed Jul 22, 2020. doi: 10.1093/ntr/ntp165.

15. Mullen KA, Manuel DG, Hawken SJ, et al. Effectiveness of a hospital-initiated smoking cessation programme: 2-year health and healthcare outcomes. *Tob Control*. 2017;26(3):293-299. https://pubmed.ncbi.nlm.nih.gov/27225016/. Accessed Jan 1, 2022. doi: 10.1136/tobaccocontrol-2015-052728.

16. Massachusetts Health & Hospital Association. Guidelines for medication for addiction treatment for opioid use disorder within the emergency department. . 2019. https://www.mhalink.org/MHADocs/MondayReport/2019/18-01-04MATguidelinesNEWFINAL.pdf.

17. MHA opioid resources for hospitals. . . https://www.mhaonline.org/docs/default-source/Resources/Opioid-Resources-for-Hospitals/webinar-series-november-14-2017.pdf?sfvrsn=2.

18. Abuse, National Institute on Drug. Overdose death rates. National Institute on Drug Abuse Web site. https://nida.nih.gov/drug-topics/trends-statistics/overdose-death-rates. Updated 2022. Accessed Jan 1, 2022.

19. D’Onofrio G, O’Connor PG, Pantalon MV, et al. Emergency Department–Initiated buprenorphine/naloxone treatment for opioid dependence. *JAMA*. 2015;313(16):1636-1644. https://www.ncbi.nlm.nih.gov/pmc/articles/PMC4527523/. Accessed Jan 1, 2022. doi: 10.1001/jama.2015.3474.

20. American College of Medical Toxicology. ACMT position statement:
Buprenorphine administration in the emergency department.

21. SAMHSA. Screening, brief
intervention and referral to treatment (SBIRT) in behavioral healthcare. 2011.

22. MedStar Health. SBIRT program off to successful start in baltimore MedStar hospitals. https://www.medstarhealth.org/news-and-publications/news/sbirt-program-off-successful-start-baltimore-medstar-hospitals Web site. https://www.medstarhealth.org/news-and-publications/news/sbirt-program-off-successful-start-baltimore-medstar-hospitals. Updated 2016. Accessed Jan 1, 2022.

23. TAGGS. Howard university hospital screening, brief intervention and referral to treatment (SBIRT) community expansion initiative https://taggs.hhs.gov/Detail/AwardDetail?arg_AwardNum=H79TI081111&arg_ProgOfficeCode=147. Accessed Jan 1, 2022.

24. Verdurmen J, Monshouwer K, van Dorsselaer S, ter Bogt T, Vollebergh W. Alcohol use and mental health in adolescents: Interactions with age and gender-findings from the dutch 2001 health behaviour in school-aged children survey. *J Stud Alcohol*. 2005;66(5):605-609. https://www.jsad.com/doi/abs/10.15288/jsa.2005.66.605. Accessed Jan 1, 2022. doi: 10.15288/jsa.2005.66.605.

25. Esser MB, Sherk A, Liu Y, et al. Deaths and years of potential life lost from excessive alcohol use - united states, 2011-2015. *MMWR Morb Mortal Wkly Rep*. 2020;69(30):981-987. https://www.cdc.gov/mmwr/volumes/69/wr/mm6939a6.htm?s_cid=mm6939a6_w#References. Accessed Jan 1, 2022. doi: 10.15585/mmwr.mm6930a1.

26. Schmidt L, Greenfield T, Mulia N. Unequal treatment racial and ethnic disparities in alcoholism treatment services. *Alcohol Res Health*. 2006;29(1):49-54. https://www.ncbi.nlm.nih.gov/pmc/articles/PMC6470902/. Accessed Jan 1, 2022.

27. Barata IA, Shandro JR, Montgomery M, et al. Effectiveness of SBIRT for alcohol use disorders in the emergency department: A systematic review. *West J Emerg Med*. 2017;18(6):1143-1152. https://www.ncbi.nlm.nih.gov/pmc/articles/PMC5654886/. Accessed Jan 1, 2022. doi: 10.5811/westjem.2017.7.34373.

28. American College of Emergency Physicians. Alcohol screening in the emergency department. *American College of Emergency Physicians*. 2017. https://www.acep.org/patient-care/policy-statements/alcohol-screening-in-the-emergency-department/. Accessed Jan 1, 2022.

29. Zun LS, Downey L, Rosen J. The effectiveness of an ED-based violence prevention program. *Am J Emerg Med*. 2006;24(1):8-13. https://pubmed.ncbi.nlm.nih.gov/16338502/. Accessed Jan 1, 2022. doi: 10.1016/j.ajem.2005.05.009.

30. National Network of Hospital-based Violence Intervention Programs. NNHVIP policy white paper hospital-based violence intervention:
Practices and policies to end the cycle of violence. . . https://static1.squarespace.com/static/5d6f61730a2b610001135b79/t/5d83c0d9056f4d4cbdb9acd9/1568915699707/NNHVIP+White+Paper.pdf.

31. Detroit Life is Valuable Everyday. Violence intervention and prevention - what is DLIVE. http://detroitlive.org/what-is-dlive/#:~:text=DLIVE%20is%20a%20Hospital%2Dbased%2C%20Community%2Dfocused%20Violence%20Intervention%20Initiative&text=Detroit%20Life%20Is%20Valuable%20Everyday%20(DLIVE)%20was%20borne%20out%20of,health%20crisis%20in%20the%20city.

32. WHO. Violence prevention approach
. https://www.who.int/violenceprevention/approach/definition/en/.

33. Fatal injury and violence data WISQARS injury center CDC. https://www.cdc.gov/injury/wisqars/fatal.html. Updated 2022. Accessed Jan 1, 2022.

34. NonFatal data WISQARS injury center CDC. https://www.cdc.gov/injury/wisqars/nonfatal.html. Updated 2022. Accessed Jan 1, 2022.

35. Kaufman E, Rising K, Wiebe DJ, Ebler DJ, Crandall ML, Delgado MK. Recurrent violent injury: Magnitude, risk factors, and opportunities for intervention from a statewide analysis. *Am J Emerg Med*. 2016;34(9):1823-1830. https://www.ncbi.nlm.nih.gov/pmc/articles/PMC4996708/. Accessed Jan 1, 2022. doi: 10.1016/j.ajem.2016.06.051.

36. Agency for Healthcare Research and Quality. Intimate partner violence screening. https://www.ahrq.gov/ncepcr/tools/healthier-pregnancy/fact-sheets/partner-violence.html. Accessed Jan 1, 2022.

37. American College of Obstetricians and Gynecologists. Intimate partner violence. https://www.acog.org/en/clinical/clinical-guidance/committee-opinion/articles/2012/02/intimate-partner-violence. Updated 2019. Accessed Jan 1, 2022.

38. Children's Hospital of Philadelphia. Why screen for intimate partner violence? because families need us to. Center for Injury Research and Prevention Web site. https://injury.research.chop.edu/blog/posts/why-screen-intimate-partner-violence-because-families-need-us. Updated 2018. Accessed Jan 1, 2022.

39. Young-Wolff KC, Kotz K, McCaw B. Transforming the health care response to intimate partner violence: Addressing “Wicked problems”. *JAMA*. 2016;315(23):2517-2518. https://doi.org/10.1001/jama.2016.4837. Accessed Jan 1, 2022. doi: 10.1001/jama.2016.4837.

40. Ramaswamy A, Ranji U, Salganicoff A. Intimate partner violence (IPV) screening and counseling services in clinical settings. KFF Web site. https://www.kff.org/womens-health-policy/issue-brief/intimate-partner-violence-ipv-screening-and-counseling-services-in-clinical-settings/. Updated 2019. Accessed Jan 1, 2022.

41. Curry SJ, Krist AH, Owens DK, et al. Screening for intimate partner violence, elder abuse, and abuse of vulnerable adults: US preventive services task force final recommendation statement. *JAMA*. 2018;320(16):1678-1687. https://jamanetwork.com/journals/jama/fullarticle/2708121. Accessed Jan 1, 2022. doi: 10.1001/jama.2018.14741.

42. Cheng D, Salimi S, Terplan M, Chisolm MS. Intimate partner violence and maternal cigarette smoking before and during pregnancy. *Obstet Gynecol*. 2015;125(2):356-362. https://www.ncbi.nlm.nih.gov/pmc/articles/PMC4866636/. Accessed Jan 1, 2022. doi: 10.1097/AOG.0000000000000609.

43. Morrison LJ, Allan R, Grunfeld A. Improving the emergency department detection rate of domestic violence using direct questioning. *J Emerg Med*. 2000;19(2):117-124. https://pubmed.ncbi.nlm.nih.gov/10903457/. Accessed Jan 1, 2022. doi: 10.1016/s0736-4679(00)00204-3.

44. Karnitschnig L, Bowker S. Intimate partner violence screening in the emergency department: A quality improvement project. *Journal of Emergency Nursing*. 2020;46(3):345-353. https://www.sciencedirect.com/science/article/pii/S0099176720300519. Accessed Jan 1, 2022. doi: 10.1016/j.jen.2020.02.008.

45. Houry D, Kaslow NJ, Kemball RS, et al. Does screening in the emergency department hurt or help victims of intimate partner violence? *Ann Emerg Med*. 2008;51(4):433-7. https://repository.upenn.edu/cgi/viewcontent.cgi?article=1124&context=spp_papers. Accessed Jan 1, 2022. doi: 10.1016/j.annemergmed.2007.11.019.

46. American College of Emergency Physicians. Domestic family violence. https://www.acep.org/imports/clinical-and-practice-management/resources/violence/domestic-family-violence/ Web site. https://www.acep.org/imports/clinical-and-practice-management/resources/violence/domestic-family-violence/. Accessed Jan 1, 2022.

47. NYC Health. New york city food standards cafeterias/cafes
. . . https://www1.nyc.gov/assets/doh/downloads/pdf/cardio/cafeterias-standards.pdf.

48. Plant-based dining grows at montefiore med center. Food Management Web site. https://www.food-management.com/healthcare/plant-based-dining-grows-montefiore-med-center. Updated 2018. Accessed Jan 1, 2022.

49. CDC. Staten island hospital puts cafeteria customers’ health first. Centers for Disease Control and Prevention Web site. https://www.cdc.gov/dhdsp/programs/srcp-ss-staten-island.htm. Updated 2020. Accessed Jan 1, 2022.

50. Hales CM, Carroll MD, Fryar CD, Ogden CL. Prevalence of obesity and severe obesity among adults : United states, 2017–2018. . 2020(360). https://stacks.cdc.gov/view/cdc/85451.

51. Lavie CJ, Milani RV, Ventura HO. Obesity and cardiovascular disease: Risk factor, paradox, and impact of weight loss. *J Am Coll Cardiol*. 2009;53(21):1925-1932. https://www.jacc.org/doi/full/10.1016/j.jacc.2008.12.068. Accessed Jan 1, 2022. doi: 10.1016/j.jacc.2008.12.068.

52. Calle EE, Thun MJ. Obesity and cancer. *Oncogene*. 2004;23(38):6365-6378. https://www.nature.com/articles/1207751. Accessed Jan 1, 2022. doi: 10.1038/sj.onc.1207751.

53. Adams KF, Schatzkin A, Harris TB, et al. Overweight, obesity, and mortality in a large prospective cohort of persons 50 to 71 years old. *New England Journal of Medicine*. 2006;355(8):763-778. https://doi.org/10.1056/NEJMoa055643. Accessed Jan 1, 2022. doi: 10.1056/NEJMoa055643.

54. Schröder H. Protective mechanisms of the mediterranean diet in obesity and type 2 diabetes. *The Journal of Nutritional Biochemistry*. 2007;18(3):149-160. https://www.sciencedirect.com/science/article/pii/S0955286306001598. Accessed Jan 1, 2022. doi: 10.1016/j.jnutbio.2006.05.006.

55. Franco M, Diez-Roux AV, Nettleton JA, et al. Availability of healthy foods and dietary patterns: The multi-ethnic study of Atherosclerosis123. *Am J Clin Nutr*. 2009;89(3):897-904. https://www.ncbi.nlm.nih.gov/pmc/articles/PMC2667662/. Accessed Jan 1, 2022. doi: 10.3945/ajcn.2008.26434.

56. Kelli HM, Kim JH, Samman Tahhan A, et al. Living in food deserts and adverse cardiovascular outcomes in patients with cardiovascular disease. *J Am Heart Assoc*. 2019;8(4):e010694. Accessed Jan 1, 2022. doi: 10.1161/JAHA.118.010694.

57. Moran A. An intervention to increase availability of healthy foods and beverages in new york city hospitals: The healthy hospital food initiative, 2010–2014. *Prev Chronic Dis*. 2016;13. https://www.cdc.gov/pcd/issues/2016/15_0541.htm. Accessed Jan 1, 2022. doi: 10.5888/pcd13.150541.

58. American Hospital Association. Screening for social needs: Guiding care teams to engage patients. 2019.

59. The National Academies of Sciences Engineering Medicine. Capturing social and behavioral domains and measures in electronic health records: Phase 2. 2014.

60. Centers for Medicare & Medicaid Services. The accountable health communities health-related social needs screening tool.

61. AAFP. Social determinants of health guide to social needs screening. 2019.

62. Nopren. Health leads screening toolkit. 2016.

63. Children's Hospital Assocation. Screening for social determinants of health. https://www.childrenshospitals.org/Issues-and-Advocacy/Population-Health/Reports/Screening-for-Social-Determinants-of-Health.

64. RWJBarnabas. RWJBarnabas health launches nation’s first universal social determinants of health program. RWJBarnabas Health Web site. https://www.rwjbh.org/blog/2020/october/rwjbarnabas-health-launches-nation-s-first-unive/. Updated 2020. Accessed Jan 1, 2022.

65. Johnson SR. Most providers still don't universally screen for social needs. Modern Healthcare Web site. https://www.modernhealthcare.com/patient-care/most-providers-still-dont-universally-screen-social-needs. Updated 2020. Accessed Jan 1, 2022.

66. Gundersen C, Ziliak JP. Food insecurity and health outcomes. *Health Affairs*. 2015;34(11):1830-1839. https://www.healthaffairs.org/doi/full/10.1377/hlthaff.2015.0645. Accessed Jan 1, 2022. doi: 10.1377/hlthaff.2015.0645.

67. Syed ST, Gerber BS, Sharp LK. Traveling towards disease: Transportation barriers to health care access. *J Community Health*. 2013;38(5):976-993. https://doi.org/10.1007/s10900-013-9681-1. doi: 10.1007/s10900-013-9681-1.

68. Thomson H, Petticrew M, Morrison D. Health effects of housing improvement: Systematic review of intervention studies. *BMJ*. 2001;323(7306):187. http://www.bmj.com/content/323/7306/187.abstract. doi: 10.1136/bmj.323.7306.187.

69. Rauh VA, Landrigan PJ, Claudio L. Housing and health: Intersection of poverty and environmental exposures. *Ann N Y Acad Sci*. 2008;1136:276-288. https://pubmed.ncbi.nlm.nih.gov/18579887/. Accessed Jan 1, 2022. doi: 10.1196/annals.1425.032.

70. Gottlieb LM, Hessler D, Long D, et al. Effects of social needs screening and in-person service navigation on child health: A randomized clinical trial. *JAMA Pediatrics*. 2016;170(11):e162521-e162521. https://doi.org/10.1001/jamapediatrics.2016.2521. Accessed Jan 1, 2022. doi: 10.1001/jamapediatrics.2016.2521.

71. Samuels-Kalow ME, Boggs KM, Cash RE, et al. Screening for health-related social needs of emergency department patients. *Annals of Emergency Medicine*. 2021;77(1):62-68. https://www.sciencedirect.com/science/article/pii/S0196064420306557. Accessed Jan 1, 2022. doi: 10.1016/j.annemergmed.2020.08.010.

72. Moon RY, Darnall RA, Feldman-Winter L, Goodstein MH, Hauck FR. SIDS and other sleep-related infant deaths: Updated 2016 recommendations for a safe infant sleeping environment. *Pediatrics*. 2016;138(5):e20162938. https://doi.org/10.1542/peds.2016-2938. Accessed Jan 1, 2022. doi: 10.1542/peds.2016-2938.

73. Walcott RL, Salm Ward TC, Ingels JB, Llewellyn NA, Miller TJ, Corso PS. A statewide hospital-based safe infant sleep initiative: Measurement of parental knowledge and behavior. *J Community Health*. 2018;43(3):534-542. https://doi.org/10.1007/s10900-017-0449-x. doi: 10.1007/s10900-017-0449-x.

74. Safe sleep. American Academy of Pediatrics Web site. http://www.aap.org/en/patient-care/safe-sleep/. Accessed Jan 1, 2022.

75. CDC. Progress in reducing risky infant sleeping positions--13 states, 1996-1997. *MMWR Morb Mortal Wkly Rep*. 1999;48(39):878-882. https://pubmed.ncbi.nlm.nih.gov/10550038/. Accessed Jan 1, 2022.

76. World HO, United Nations Children's Fund, (UNICEF). *Implementation guidance: Protecting, promoting and supporting breastfeeding in facilities providing maternity and newborn services: The revised baby-friendly hospital initiative.* Geneva: World Health Organization; 2018. https://apps.who.int/iris/handle/10665/272943.

77. Philipp BL, Merewood A, Miller LW, et al. Baby-friendly hospital initiative improves breastfeeding initiation rates in a US hospital setting. *Pediatrics*. 2001;108(3):677-681. https://pubmed.ncbi.nlm.nih.gov/11533335/. Accessed Jan 1, 2022. doi: 10.1542/peds.108.3.677.

78. NEOVITA Study Group. Timing of initiation, patterns of breastfeeding, and infant survival: Prospective analysis of pooled data from three randomised trials. *The Lancet Global Health*. 2016;4(4):e266-e275. https://www.sciencedirect.com/science/article/pii/S2214109X16000401. Accessed Jan 1, 2022. doi: 10.1016/S2214-109X(16)00040-1.

79. Yan J, Liu L, Zhu Y, Huang G, Wang PP. The association between breastfeeding and childhood obesity: A meta-analysis. *BMC Public Health*. 2014;14. https://www.ncbi.nlm.nih.gov/pmc/articles/PMC4301835/. Accessed Jan 1, 2022. doi: 10.1186/1471-2458-14-1267.

80. Schwarz, Eleanor B., MD, MS, Nothnagle, Melissa, MD, MSC. The maternal health benefits of breastfeeding. *American family physician*. 2015;91(9):602-604. https://www.clinicalkey.es/playcontent/1-s2.0-S0002838X15301003.

81. World Health Organization Division of Child Health, and Development. Evidence for the ten steps to successful breastfeeding. . 1998. https://apps.who.int/iris/handle/10665/64877.

82. Pérez-Escamilla R, Martinez JL, Segura-Pérez S. Impact of the baby-friendly hospital initiative on breastfeeding and child health outcomes: A systematic review. *Matern Child Nutr*. 2016;12(3):402-417. Accessed Jan 1, 2022. doi: 10.1111/mcn.12294.

83. American College of Obstetricians and Gynecologists. Immediate postpartum long-acting reversible contraception. https://www.acog.org/clinical/clinical-guidance/committee-opinion/articles/2016/08/immediate-postpartum-long-acting-reversible-contraception Web site. https://www.acog.org/en/clinical/clinical-guidance/committee-opinion/articles/2016/08/immediate-postpartum-long-acting-reversible-contraception. Updated 2020. Accessed Jan 1, 2022.

84. Michelle moniz: Q&A on coverage for immediate postpartum long-acting reversible contraception. Institute for Healthcare Policy & Innovation University of Michigan Web site. https://ihpi.umich.edu/news/michelle-moniz-qa-coverage-immediate-postpartum-long-acting-reversible-contraception. Updated 2018. Accessed Jan 1, 2022.

85. Thagard AS, Napolitano PG, Bryant AS. The role of extremes in interpregnancy interval in women at increased risk for adverse obstetric outcomes due to health disparities:  A literature review. *Curr Womens Health Rev*. 2018;14(3):242-250. Accessed Jan 1, 2022. doi: 10.2174/1573404813666170323154244.

86. Hanley GE, Hutcheon JA, Kinniburgh BA, Lee L. Interpregnancy interval and adverse pregnancy outcomes: An analysis of successive pregnancies. *Obstet Gynecol*. 2017;129(3):408-415. Accessed Jan 1, 2022. doi: 10.1097/AOG.0000000000001891.

87. Conde-Agudelo A, Belizán JM. Maternal morbidity and mortality associated with interpregnancy interval: Cross sectional study. *BMJ*. 2000;321(7271):1255-1259. Accessed Jan 1, 2022. doi: 10.1136/bmj.321.7271.1255.

88. McKinney D, House M, Chen A, Muglia L, DeFranco E. The influence of interpregnancy interval on infant mortality. *Am J Obstet Gynecol*. 2017;216(3):316.e1-316.e9. Accessed Jan 1, 2022. doi: 10.1016/j.ajog.2016.12.018.

89. Brunson MR, Klein DA, Olsen CH, Weir LF, Roberts TA. Postpartum contraception: Initiation and effectiveness in a large universal healthcare system. *Am J Obstet Gynecol*. 2017;217(1):55.e1-55.e9. Accessed Jan 1, 2022. doi: 10.1016/j.ajog.2017.02.036.

90. Fulmer T, Pelton L. What is an age-friendly health system? | IHI - institute for healthcare improvement. http://www.ihi.org/Engage/Initiatives/Age-Friendly-Health-Systems/Pages/default.aspx Web site. http://www.ihi.org:80/Engage/Initiatives/Age-Friendly-Health-Systems/Pages/default.aspx. Accessed Jan 1, 2022.

91. IHI - Institute for Healthcare Improvement. Health systems recognized by IHI. http://www.ihi.org:80/Engage/Initiatives/Age-Friendly-Health-Systems/Pages/Background.aspx. Accessed Jan 1, 2022.

92. Bergen G, Stevens MR, Burns ER. Falls and fall injuries among adults aged ≥65 years - united states, 2014. *MMWR Morb Mortal Wkly Rep*. 2016;65(37):993-998. Accessed Jan 1, 2022. doi: 10.15585/mmwr.mm6537a2.

93. Mahoney JE, Palta M, Johnson J, et al. Temporal association between hospitalization and rate of falls after discharge. *Archives of Internal Medicine*. 2000;160(18):2788-2795. https://doi.org/10.1001/archinte.160.18.2788. Accessed Jan 1, 2022. doi: 10.1001/archinte.160.18.2788.

94. Keeler E, Guralnik JM, Tian H, Wallace RB, Reuben DB. The impact of functional status on life expectancy in older persons. *J Gerontol A Biol Sci Med Sci*. 2010;65A(7):727-733. https://www.ncbi.nlm.nih.gov/pmc/articles/PMC2884085/. Accessed Jan 1, 2022. doi: 10.1093/gerona/glq029.

95. ASTHO. Community health needs assessment consensus statement. https://www.astho.org/Programs/Access/Community-Health-Needs-Assessment/Consensus-Statement/.

96. PRWeb. Health planning council of northeast florida and local hospitals form community benefit partnership to address local health issues. Cision PRWeb Web site. https://www.prweb.com/releases/2013/2/prweb10422869.htm. Updated 2013. Accessed Jan 1, 2022.

97. Cincinnati CHNA report. https://healthcollab.org/wp-content/uploads/2016/02/Cincinnati-CHNA-Report-2016-FINAL.pdf. Updated 2016.

98. Pennel CL, McLeroy KR, Burdine JN, Matarrita-Cascante D. Nonprofit hospitals’ approach to community health needs assessment. *https://doi.org/10.2105/AJPH.2014.302286*. 2015. https://ajph.aphapublications.org/doi/pdf/10.2105/AJPH.2014.302286. Accessed Jan 1, 2022. doi: 10.2105/AJPH.2014.302286.

99. Cedars-Sinai Staff. Barbershop-based study lowers blood pressure in black men. Cedars-Sinai Web site. https://www.cedars-sinai.org/blog/barbershop-based-study-lowers-blood-pressure-black-men.html. Updated 2018. Accessed Jan 1, 2022.

100. Herbers K. Barbershops targeted to improve health of black men. Vanderbilt University Web site. https://news.vumc.org/2020/02/18/barbershops-targeted-to-improve-health-of-black-men/. Updated 2020. Accessed Jan 1, 2022.

101. Estimated hypertension prevalence, treatment, and control among U.S. adults. . 2021. https://millionhearts.hhs.gov/data-reports/hypertension-prevalence.html#:~:text=Nearly%201%20out%20of%202,modifications%20only%20(21%20million).

102. Lackland DT. Racial differences in hypertension: Implications for high blood pressure management. *The American Journal of the Medical Sciences*. 2014;348(2):135-138. https://www.sciencedirect.com/science/article/pii/S0002962915302585. Accessed Jan 1, 2022. doi: 10.1097/MAJ.0000000000000308.

103. Kong BW. Community-based hypertension control programs that work. *J Health Care Poor Underserved*. 1997;8(4):409-415. Accessed Jan 1, 2022. doi: 10.1353/hpu.2010.0031.

104. Victor RG, Ravenell JE, Freeman A, et al. Effectiveness of a barber-based intervention for improving hypertension control in black men: The BARBER-1 study: A cluster randomized trial. *Arch Intern Med*. 2011;171(4):342-350. https://doi.org/10.1001/archinternmed.2010.390. doi: 10.1001/archinternmed.2010.390.

105. FORTMANN SP, WINKLEBY MA, FLORA JA, HASKELL WL, TAYLOR CB. Effect of long-term community health education on blood pressure and hypertension control: The stanford five-city project. *American Journal of Epidemiology*. 1990;132(4):629-646. https://doi.org/10.1093/oxfordjournals.aje.a115705. Accessed Jan 1, 2022. doi: 10.1093/oxfordjournals.aje.a115705.

106. Kong BW. Community programs to increase hypertension control. *J Natl Med Assoc*. 1989;81(Suppl):13-16. https://www.ncbi.nlm.nih.gov/pmc/articles/PMC2625778/. Accessed Jul 22, 2020.

107. Unger T, Borghi C, Charchar F, et al. 2020 international society of hypertension global hypertension practice guidelines. *Hypertension*. 2020;75(6):1334-1357. https://www.ahajournals.org/doi/10.1161/HYPERTENSIONAHA.120.15026. Accessed Jan 1, 2022. doi: 10.1161/HYPERTENSIONAHA.120.15026.

108. Rehm CD, Marquez ME, Spurrell-Huss E, Hollingsworth N, Parsons AS. Lessons from launching the diabetes prevention program in a large integrated health care delivery system: A case study. *Popul Health Manag*. 2017;20(4):262-270. https://www.ncbi.nlm.nih.gov/pmc/articles/PMC5564042/. Accessed Jan 1, 2022. doi: 10.1089/pop.2016.0109.

109. Wright J. West virginia preventive health and health services (PHHS) block grant success story
partnering to prevent diabetes in west virginia: Diabetes prevention programs in hospitals. . 2017.

110. Knowler WC, Barrett-Connor E, Fowler SE, et al. Reduction in the incidence of type 2 diabetes with lifestyle intervention or metformin. *N Engl J Med*. 2002;346(6):393-403. Accessed Jan 1, 2022. doi: 10.1056/NEJMoa012512.

111. Albright AL, Gregg EW. Preventing type 2 diabetes in communities across the U.S.: The national diabetes prevention program. *Am J Prev Med*. 2013;44(4 Suppl 4):346. Accessed Jan 1, 2022. doi: 10.1016/j.amepre.2012.12.009.

112. Home visiting programs welcome baby program. . . https://www.first5la.org/home-visiting-programs/. Accessed Jan 1, 2022.

113. Nurse-family partnership - social healthcare programs that work. Social Programs that Work Web site. https://evidencebasedprograms.org/programs/nurse-family-partnership/. Accessed Jan 1, 2022.

114. Evelyn's story. . . https://www.nursefamilypartnership.org/stories/evelyn/. Accessed Jan 1, 2022.

115. Duffee JH, Mendelsohn AL, Kuo AA, et al. Early childhood home visiting. *Pediatrics*. 2017;140(3):e20172150. https://doi.org/10.1542/peds.2017-2150. Accessed Jan 1, 2022. doi: 10.1542/peds.2017-2150.

116. PASOS (STEPS) greenville health system. https://www.aha.org/system/files/2018-10/2018-ghs-pasos-chw-case-study.pdf.

117. IMPaCT home - penn center for community health workers. Penn Center for Community Health Workers Web site. https://chw.upenn.edu/. Accessed Jan 1, 2022.

118. Weiner S. Little-known health workers, coming soon to a hospital near you. AAMC Web site. https://www.aamc.org/news-insights/little-known-health-workers-coming-soon-hospital-near-you.

119. What we do – NACHW. NACHW Web site. https://nachw.org/about/. Accessed Jan 1, 2022.

120. Fedder DO, Chang RJ, Curry S, Nichols G. The effectiveness of a community health worker outreach program on healthcare utilization of west baltimore city medicaid patients with diabetes, with or without hypertension. *Ethn Dis*. 2003;13(1):22-27. Accessed Jun 28, 2021.

121. Sharma N, Harris E, Lloyd J, Mistry SK, Harris M. Community health workers involvement in preventative care in primary healthcare: A systematic scoping review. *BMJ Open*. 2019;9(12):e031666. https://bmjopen.bmj.com/content/9/12/e031666. Accessed Jun 28, 2021. doi: 10.1136/bmjopen-2019-031666.

122. Kangovi S, Mitra N, Grande D, et al. Patient-centered community health worker intervention to improve posthospital outcomes: A randomized clinical trial. *JAMA Internal Medicine*. 2014;174(4):535-543. https://doi.org/10.1001/jamainternmed.2013.14327. Accessed Jun 28, 2021. doi: 10.1001/jamainternmed.2013.14327.

123. The impact of community health workers. AHA Web site. https://www.aha.org/advocacy-webinar-recording/2017-06-07-impact-community-health-workers. Accessed Jan 1, 2022.

124. Hospital school partnerships benefit students and the community. Childrens Hospitals Web site. https://www.childrenshospitals.org/Newsroom/Childrens-Hospitals-Today/Issue-Archive/Issues/Summer-2016/Articles/Hospital-School-Partnerships-Benefit-Students-and-the-Community.

125. Literacy: A path to lifelong health. Cincinatti Children's Web site. https://www.cincinnatichildrens.org/giving/how-you-help/stories/ftc/archives/2017/spring/new-approach.

126. Allison MA, Attisha E, Lerner M, et al. The link between school attendance and good health. *Pediatrics*. 2019;143(2):e20183648. https://doi.org/10.1542/peds.2018-3648. Accessed Jan 1, 2022. doi: 10.1542/peds.2018-3648.

127. DeWalt DA, Berkman ND, Sheridan S, Lohr KN, Pignone MP. Literacy and health outcomes. *Journal of General Internal Medicine*. 2004;19(12):1228-1239. https://doi.org/10.1111/j.1525-1497.2004.40153.x. doi: 10.1111/j.1525-1497.2004.40153.x.

128. Molla MT, Madans JH, Wagener DK. Differentials in adult mortality and activity limitation by years of education in the united states at the end of the 1990s. *Population and Development Review*. 2004;30(4):625-646. https://onlinelibrary.wiley.com/doi/abs/10.1111/j.1728-4457.2004.00035.x. Accessed Jan 1, 2022. doi: 10.1111/j.1728-4457.2004.00035.x.

129. Braunstein S, Lavizzo-Mourey R. How the health and community development sectors are combining forces to improve health and well-being. *Health Affairs*. 2011;30(11):2042-2051. https://www.healthaffairs.org/doi/full/10.1377/hlthaff.2011.0838. Accessed Jan 1, 2022. doi: 10.1377/hlthaff.2011.0838.

130. Farmers market locations. Kaiser Permanente Web site. https://healthy.kaiserpermanente.org/health-wellness/farmers-markets/locations. Accessed Jan 1, 2022.

131. How hospitals are boosting healthy food access in vulnerable communities. AHA News Web site. https://www.aha.org/news/insights-and-analysis/2019-01-22-how-hospitals-are-boosting-healthy-food-access-vulnerable. Updated 2019. Accessed Jan 1, 2022.

132. Food security and nutrition assistance. USDA Web site. https://www.ers.usda.gov/data-products/ag-and-food-statistics-charting-the-essentials/food-security-and-nutrition-assistance/.

133. Beaulac J, Kristjansson E, Cummins S. A systematic review of food deserts, 1966-2007. *Prev Chronic Dis*. 2009;6(3). https://www.ncbi.nlm.nih.gov/pmc/articles/PMC2722409/. Accessed Jan 1, 2022.

134. Piontak JR, Schulman MD. Food insecurity in rural america. *Contexts*. 2014;13(3):75-77. https://doi.org/10.1177/1536504214545766. Accessed Jan 1, 2022. doi: 10.1177/1536504214545766.

135. Gittelsohn J, Rowan M, Gadhoke P. Interventions in small food stores to change the food environment, improve diet, and reduce risk of chronic disease. *Prev Chronic Dis*. 2012;9. https://www.ncbi.nlm.nih.gov/pmc/articles/PMC3359101/. Accessed Jan 1, 2022.

136. Social determinants of health series: Housing and the role of hospitals. AHA Web site. https://www.aha.org/ahahret-guides/2017-08-22-social-determinants-health-series-housing-and-role-hospitals. Accessed Jan 1, 2022.

137. Healthy home program
. Childrens Mercy Web site. https://www.childrensmercy.org/in-the-community/environmental-health-program/healthy-home-program/.

138. Affordable housing. Nationwide Children's Web site. https://www.nationwidechildrens.org/about-us/population-health-and-wellness/healthy-neighborhoods-healthy-families/affordable-housing. Accessed Jan 1, 2022.

139. Nationwide children’s hospital named among “Accelerating investments for healthy communities” participants for efforts to increase affordable housing. Nationwide Children's Web site. https://www.nationwidechildrens.org/newsroom/news-releases/2019/02/aihc-phase-ii. Updated 2019. Accessed Jan 1, 2022.

140. Kuehn BM. Hospitals turn to housing to help homeless patients. *JAMA*. 2019;321(9):822-824. https://doi.org/10.1001/jama.2018.21476. Accessed Jan 1, 2022. doi: 10.1001/jama.2018.21476.

141. New jersey housing and mortgage finance agency. https://www.nj.gov/dca/hmfa/media/news/2019/approved/20190710.html.

142. Watson NE, Steffen BL, Martin M, Vandenbroucke DA. Worst case housing needs: 2019 report to congress . 2020.

143. Krieger J, Higgins DL. Housing and health: Time again for public health action. *Am J Public Health*. 2002;92(5):758-768. https://www.ncbi.nlm.nih.gov/pmc/articles/PMC1447157/. Accessed Jan 1, 2022.

144. Baggett TP, Hwang SW, O'Connell JJ, et al. Mortality among homeless adults in boston: Shifts in causes of death over a 15-year period. *JAMA Intern Med*. 2013;173(3):189-195. https://www.ncbi.nlm.nih.gov/pmc/articles/PMC3713619/. Accessed Jan 1, 2022. doi: 10.1001/jamainternmed.2013.1604.

145. Fineman RW. The shortest path isolation index: A new measure for individual-level residential segregation. *Sociological Methods & Research*. 2020;49(3):742-777. https://doi.org/10.1177/0049124118769097. Accessed Jan 1, 2022. doi: 10.1177/0049124118769097.

146. Allen R, Burgess S, Davidson R, Windmeijer F. More reliable inference for the dissimilarity index of segregation. *Econom J*. 2015;18(1):40-66. https://www.ncbi.nlm.nih.gov/pmc/articles/PMC5054828/. Accessed Jan 1, 2022. doi: 10.1111/ectj.12039.

147. Cohen M, Pettit KLS. Guide to measuring neighborhood change to understand and prevent displacement. *National Neighborhood Indicators Partnership*. 2019.

148. Zottarelli LK, Sharif HO, Xu X, Sunil TS. Effects of social vulnerability and heat index on emergency medical service incidents in san antonio, texas, in 2018. *J Epidemiol Community Health*. 2021;75(3):271. http://jech.bmj.com/content/75/3/271.abstract. doi: 10.1136/jech-2019-213256.

149. Fletcher JM, Andreyeva T, Busch SH. Assessing the effect of changes in housing costs on food insecurity. *Journal of Children and Poverty*. 2009;15(2):79-93. https://doi.org/10.1080/10796120903310541. Accessed Jan 1, 2022. doi: 10.1080/10796120903310541.

150. Surratt HL, O'Grady CL, Levi-Minzi MA, Kurtz SP. Medication adherence challenges among HIV positive substance abusers: The role of food and housing insecurity. *AIDS Care*. 2015;27(3):307-314. https://doi.org/10.1080/09540121.2014.967656. Accessed Jan 1, 2022. doi: 10.1080/09540121.2014.967656.

151. Children’s mercy kansas city hospital is improving health by improving housing. Office of Policy Development and Research Web site. https://www.huduser.gov/portal/casestudies/study-100118.html.

152. Washburn L. St. joseph's health to build unique, affordable housing in paterson. North Jersey Media Group Web site. https://www.northjersey.com/story/news/2019/07/10/st-josephs-health-develop-affordable-housing-paterson/1687588001/. Updated 2018. Accessed Jan 1, 2022.

153. Community development and health: Alignment opportunities for CDFIs and hospitals. *Unversity of New Hampshire Carey School of Public Policy Public Health Institute*. 2015.

154. Asante-Muhammad D, Chase D, Richardson J. NAACP opportunity and diversity report card: The healthcare industry. http://arks.princeton.edu/ark:/88435/dsp014q77fv22s. Updated 2015.

155. Gurkin C. Strategies to increase representation of women and Minorities—Testimony before the committee on financial services, house of representatives. . 2019. https://corpgov.law.harvard.edu/2019/06/27/strategies-to-increase-representation-of-women-and-minorities-testimony-before-the-committee-on-financial-services-house-of-representatives/. Accessed Jan 1, 2022.

156. Totten M. How and why to increase board diversity | AHA trustee services. https://trustees.aha.org/articles/916-how-and-why-to-increase-board-diversity Web site. https://trustees.aha.org/articles/916-how-and-why-to-increase-board-diversity. Accessed Jan 1, 2022.

157. Livingston S. Fostering diversity for the next generation of healthcare leaders. Modern Healthcare Web site. https://www.modernhealthcare.com/article/20181013/NEWS/181019970/fostering-diversity-for-the-next-generation-of-healthcare-leaders. Updated 2018. Accessed Jan 1, 2022.

158. AHA Trustee Services. AHA 2019 national health care governance survey report. https://trustees.aha.org/aha-2019-national-health-care-governance-survey-report. Accessed Jan 1, 2022.

159. Flores K, Combs G. Minority representation in healthcare: Increasing the number of professionals through focused recruitment. *Hospital Topics*. 2013;91(2):25-36. https://doi.org/10.1080/00185868.2013.793556. Accessed Jan 1, 2022. doi: 10.1080/00185868.2013.793556.

160. Henkel G. Does U.S. healthcare need more diverse leadership? https://www.the-hospitalist.org/hospitalist/article/121639/does-us-healthcare-need-more-diverse-leadership. Updated 2016.

161. Herrin J, Harris KG, Spatz E, Cobbs-Lomax D, Allen S, León T. Hospital leadership diversity and strategies to advance health equity. *The Joint Commission Journal on Quality and Patient Safety*. 2018;44(9):545-551. https://www.sciencedirect.com/science/article/pii/S1553725017304543. Accessed Jan 1, 2022. doi: 10.1016/j.jcjq.2018.03.008.

162. Living wage calculation for baltimore city, maryland https://livingwage.mit.edu/counties/24510 Web site.

163. Minimum wage. US Department of Labor Wage and Hour Division Web site. https://www.dol.gov/agencies/whd/minimum-wage.

164. Leigh JP, Leigh W, Du J. Minimum wages and public health: A literature review. . 2018. https://papers.ssrn.com/abstract=3176217. Accessed Jan 1, 2022. doi: 10.2139/ssrn.3176217.

165. Komro KA, Livingston MD, Markowitz S, Wagenaar AC. The effect of an increased minimum wage on infant mortality and birth weight. *Am J Public Health*. 2016;106(8):1514-1516. https://www.ncbi.nlm.nih.gov/pmc/articles/PMC4940666/. Accessed Jan 1, 2022. doi: 10.2105/AJPH.2016.303268.

166. Himmelstein KEW, Venkataramani AS. Economic vulnerability among US female health care workers: Potential impact of a $15-per-hour minimum wage. *American journal of public health (1971)*. 2019;109(2):198-205. https://www.ncbi.nlm.nih.gov/pubmed/30571300. doi: 10.2105/AJPH.2018.304801.

167. Modern Healthcare. A living wage for healthcare workers is essential to achieve health equity. Institute for Healthcare Improvement Web site. http://www.ihi.org:80/about/news/Pages/A-Living-Wage-for-Healthcare-Workers-Is-Essential-to-Achieve-Health-Equity.aspx. Accessed Jan 1, 2022.

168. Supplier diversity and inclusion program. UPMC Web site. https://www.upmc.com/about/supply-chain/supplier-diversity-program. Accessed Jan 1, 2022.

169. About us - supplier diversity program. Mayo Clinic Web site. https://www.mayoclinic.org/about-mayo-clinic/supplier-information/diversity. Accessed Jan 1, 2022.

170. Local, small, minority, women-owned, HUBZone and veteran-owned business development progra Johns Hopkins University - University Financial Services Web site. https://finance.jhu.edu/depts/procurement/procurement_policies/minority_program_jhu.html.

171. Supplier diversity. Kaiser Permanente Web site. http://supplier.kp.org/supplierdiversity.html.

172. Kondo N, Sembajwe G, Kawachi I, van Dam R,M., Subramanian SV, Yamagata Z. Income inequality, mortality, and self rated health: Meta-analysis of multilevel studies. *BMJ*. 2009;339:b4471. http://www.bmj.com/content/339/bmj.b4471.abstract. doi: 10.1136/bmj.b4471.

173. Perry VG. A loan at last? race and racism in mortgage lending. In: Johnson GD, Thomas KD, Harrison AK, Grier SA, eds. *Race in the marketplace: Crossing critical boundaries.* Cham: Springer International Publishing; 2019:173-192. https://doi.org/10.1007/978-3-030-11711-5_11. 10.1007/978-3-030-11711-5_11.

174. Chetty R, Hendren N, Jones MR, Porter SR. Race and economic opportunity in the united states: An intergenerational perspective. *The Quarterly Journal of Economics*. 2020;135(2):711-783. https://doi.org/10.1093/qje/qjz042. Accessed Jan 1, 2022. doi: 10.1093/qje/qjz042.

175. Chetty R, Stepner M, Abraham S, et al. The association between income and life expectancy in the united states, 2001–2014. *JAMA*. 2016;315(16):1750-1766. https://www.ncbi.nlm.nih.gov/pmc/articles/PMC4866586/. Accessed Jan 1, 2022. doi: 10.1001/jama.2016.4226.

176. On views of race and inequality, blacks and whites are worlds apart - demographic trends and economic well-being. . 2016. https://www.pewresearch.org/social-trends/2016/06/27/1-demographic-trends-and-economic-well-being/. Accessed Jan 1, 2022.

177. Bor J, Cohen GH, Galea S. Population health in an era of rising income inequality: USA, 1980–2015. *The Lancet*. 2017;389(10077):1475-1490. https://www.sciencedirect.com/science/article/pii/S0140673617305718. Accessed Jan 1, 2022. doi: 10.1016/S0140-6736(17)30571-8.

178. Guide for employer-supported childcare. International Finance Corporation World Bank Group Web site. https://www.ifc.org/wps/wcm/connect/Topics_Ext_Content/IFC_External_Corporate_Site/Gender+at+IFC/Resources/Guide+for+Employer-Supported+Childcare. Accessed Jan 1, 2022.

179. Child care center at UH parma medical center. University Hospitals Web site. https://www.uhhospitals.org/locations/uh-parma-medical-center/services/child-care-center.

180. On-site early childhood education programs. Stanford - Cardinal at Work Web site. https://cardinalatwork.stanford.edu/benefits-rewards/worklife/children-family/site-early-childhood-education-programs. Accessed Jan 1, 2022.

181. Health implications of children in child care centres part A: Canadian trends in child care, behaviour and developmental outcomes. *Paediatr Child Health*. 2008;13(10):863-867. https://www.ncbi.nlm.nih.gov/pmc/articles/PMC2603507/. Accessed Jan 1, 2022.

182. Day JC, Christnacht C. Women hold 76% of all health care jobs, gaining in higher-paying occupations. United States Census Bureau Web site. https://www.census.gov/library/stories/2019/08/your-health-care-in-womens-hands.html. Updated 2019.

183. Chevalier A, Viitanen TK. The causality between female labour force participation and the availability of childcare. *Applied Economics Letters*. 2010. https://www.tandfonline.com/doi/abs/10.1080/13504850210138469. Accessed Jan 1, 2022. doi: 10.1080/13504850210138469.

184. Donoghue EA, Lieser D, DelConte B, et al. Quality early education and child care from birth to kindergarten. *Pediatrics*. 2017;140(2):e20171488. https://doi.org/10.1542/peds.2017-1488. Accessed Jan 1, 2022. doi: 10.1542/peds.2017-1488.

185. Conti G, Heckman JJ, Pinto R. The effects of two influential early childhood interventions on health and healthy behaviour. *The Economic Journal*. 2016;126(596):F28-F65. https://onlinelibrary.wiley.com/doi/abs/10.1111/ecoj.12420. Accessed Jan 1, 2022. doi: 10.1111/ecoj.12420.

186. Gormley WT, Gayer T, Phillips D, Dawson B. The effects of universal pre-K on cognitive development. *Dev Psychol*. 2005;41(6):872-884. Accessed Jan 1, 2022. doi: 10.1037/0012-1649.41.6.872.

187. Families first coronavirus response act: Employee paid leave rights. US Department of Labor Wage and Hour Division Web site. https://www.dol.gov/agencies/whd/pandemic/ffcra-employee-paid-leave.

188. University of rochester short-term disability plan-at-A-glance. *University of Rochester*.

189. 15 healthcare employers who offer paid sick leave in the northern US. https://transparency.kununu.com/15-healthcare-employers-who-offer-paid-sick-leave-in-the-northern-us/.

190. Paid time off | BJC employee PTO benefits. BJC Total Rewards Web site. https://www.bjctotalrewards.org/Work-Life/Paid-Time-Off.

191. DeRigne L, Stoddard-Dare P, Quinn L. Workers without paid sick leave less likely to take time off for illness or injury compared to those with paid sick leave. *Health Affairs*. 2016;35(3):520-527. https://www.healthaffairs.org/doi/full/10.1377/hlthaff.2015.0965. Accessed Jan 1, 2022. doi: 10.1377/hlthaff.2015.0965.

192. Peipins LA, Soman A, Berkowitz Z, White MC. The lack of paid sick leave as a barrier to cancer screening and medical care-seeking: Results from the national health interview survey. *BMC Public Health*. 2012;12(1):520. https://doi.org/10.1186/1471-2458-12-520. doi: 10.1186/1471-2458-12-520.

193. DeRigne L, Stoddard-Dare P, Collins C, Quinn L. Paid sick leave and preventive health care service use among U.S. working adults. *Preventive Medicine*. 2017;99:58-62. https://www.sciencedirect.com/science/article/pii/S0091743517300440. Accessed Jan 1, 2022. doi: 10.1016/j.ypmed.2017.01.020.

194. Asfaw A, Pana-Cryan R, Rosa R. Paid sick leave and nonfatal occupational injuries. *Am J Public Health*. 2012;102(9):e59-e64. https://ajph.aphapublications.org/doi/full/10.2105/ajph.2011.300482. Accessed Jan 1, 2022. doi: 10.2105/AJPH.2011.300482.

195. Paid sick leave H-440.823. AMA Policy Finder Web site. https://policysearch.ama-assn.org/policyfinder/detail/H-440.823?uri=%2FAMADoc%2FHOD-440.823.xml.

196. Statement of policy paid sick leave. 2018.

197. Curtis JL, Rukavina M. 5 ways methodist can repair damage from aggressive debt collections. MLK50: Justice Through Journalism Web site. http://mlk50.com/2019/07/19/5-ways-methodist-can-repair-damage-from-aggressive-debt-collections/. Updated 2019. Accessed Jan 1, 2022.

198. Patient billing guidelines. AHA Web site. https://www.aha.org/standardsguidelines/2020-10-15-patient-billing-guidelines. Updated 2020. Accessed Jan 1, 2022.

199. Financial assistance policies. St Luke's University Health Network Finance Web site. https://www.slhn.org/billpay/policies-and-procedures/financial-assistance-policies. Accessed Jan 1, 2022.

200. Kliff S. With medical bills skyrocketing, more hospitals are suing for payment. *The New York Times*. Nov 8, 2019. Available from: https://www.nytimes.com/2019/11/08/us/hospitals-lawsuits-medical-debt.html.

201. HERMAN PM, RISSI JJ, WALSH ME. Health insurance status, medical debt, and their impact on access to care in arizona. *American journal of public health (1971)*. 2011;101(8):1437-1443. https://www.ncbi.nlm.nih.gov/pubmed/21680916. doi: 10.2105/AJPH.2010.300080.

202. O'Toole TP, Arbelaez JJ, Lawrence RS. Medical debt and aggressive debt restitution practices. *Journal of General Internal Medicine*. 2004;19(7):772-778. https://onlinelibrary.wiley.com/doi/abs/10.1111/j.1525-1497.2004.30099.x. Accessed Jan 1, 2022. doi: 10.1111/j.1525-1497.2004.30099.x.

203. Hospital field supports billing & collection guidelines that treat patients with dignity and respect. *AHA*. 2012. https://www.aha.org/press-releases/2012-05-30-hospital-field-supports-billing-collection-guidelines-treat-patients. Accessed Jan 1, 2022.

204. The successful reentry project: Working towards justice, dignity and redemption. *National Association for the Advancement of Colored People*.

205. Meyer H. Some hospitals aim to improve community health by hiring ex-offenders. Modern Healthcare Web site. https://www.modernhealthcare.com/providers/some-hospitals-aim-improve-community-health-hiring-ex-offenders. Updated 2020. Accessed Jan 1, 2022.

206. Johns hopkins university health system. https://hospitaltoolkits.org/workforce/case-studies/johns-hopkins-university-health-system/.

207. Christman A, Rodriguez MN. Research supports fair chance policies. National Employment Law Project Web site. https://www.nelp.org/publication/research-supports-fair-chance-policies/. Updated 2016. Accessed Jan 1, 2022.

208. Pager D. The mark of a criminal record. *American Journal of Sociology*. 2003;108(5):937-975. https://www.journals.uchicago.edu/doi/10.1086/374403. Accessed Jan 1, 2022. doi: 10.1086/374403.

209. Environmental sustainability in hospitals: The value of efficiency. *AHA*. 2014. https://www.aha.org/ahahret-guides/2014-05-28-environmental-sustainability-hospitals-value-efficiency. Accessed Jan 1, 2022.

210. Memorial hermann health system. Houston Hospitals, Institutes & Centers Web site. http://www.memorialhermann.org/. Updated 2018. Accessed Jan 1, 2022.

211. Wentworth-douglass hospital: Healthcare with heart. Wentworth-Douglass Hospital Web site. https://www.wdhospital.org/wdh.

212. Carilion clinic. Carilion Clinic Web site. https://www.carilionclinic.org/.

213. Sullivan county community hospital. Sullivan County Community Hospital Web site. http://schosp.com/. Accessed Jan 1, 2022.

214. Climate effects on health. CDC Web site. https://www.cdc.gov/climateandhealth/effects/default.htm. Updated 2021. Accessed Jan 1, 2022.

215. The social dimensions discussion draft. *WHO*.

216. Islam SN, Winkel J. Climate change and social inequality. *UN*. 2017.

217. Managing air quality - human health, environmental and economic assessments. United States Environmental Protection Agency Web site. https://www.epa.gov/air-quality-management-process/managing-air-quality-human-health-environmental-and-economic. Updated 2015. Accessed Jan 1, 2022.

218. Water sanitation health publications. https://www.who.int/water_sanitation_health/publications/whoiwa/en/.

219. Mikati I, Benson AF, Luben TJ, Sacks JD, Richmond-Bryant J. Disparities in distribution of particulate matter emission sources by race and poverty status. https://doi.org/10.2105/AJPH.2017.304297 Web site. https://ajph.aphapublications.org/doi/pdf/10.2105/AJPH.2017.304297. Updated 2018. Accessed Jan 1, 2022.

220. Do no harm: America's leading health systems commit to reducing emissions. We Are Still In Web site. https://www.wearestillin.com/news/do-no-harm-americas-leading-health-systems-commit-reducing-emissions. Accessed Jan 1, 2022.

221. LEED rating system. U.S. Green Building Council Web site. https://www.usgbc.org/leed.

stylefix
